# Parent reported barriers and facilitators to support services for autistic children in Aotearoa New Zealand

**DOI:** 10.1177/13623613231168240

**Published:** 2023-04-27

**Authors:** Carla Wallace-Watkin, Jeff Sigafoos, Lisa Woods, Hannah Waddington

**Affiliations:** Victoria University of Wellington, New Zealand

**Keywords:** barriers, facilitators, parent perspectives, support services, New Zealand

## Abstract

We surveyed New Zealand parents of autistic children to identify their perceived barriers and facilitators to accessing support services, including the influence of a family’s level of financial resourcing. A total of 173 completed surveys were analysed. Service pathway factors were the greatest barrier experienced by participants, whereas facilitators related to providers tended to be most helpful. Lower family income was associated with a higher number of reported barriers. Both lower family income and having a child who identified their gender as non-binary were predictive of rating the extent of barriers higher. Parents of younger or of non-speaking autistic children on average reported a greater number of facilitators. Implications of these results for support service delivery and potential areas for future research are discussed.

**Lay abstract:**

Parents might have problems in getting support services for their autistic child due to certain barriers. However, there might also be things that can ease or facilitate parents’ access to support services. In this study, New Zealand parents were asked about their experiences in getting support services for their autistic child. We also looked at differences in reported barriers and facilitators based on several demographic factors with a focus on family level of financial resourcing. A total of 173 parents completed a survey. The results suggested that parents experienced several barriers, particularly related to service pathways. Facilitators were also experienced, predominantly related to providers. Financial resourcing predicted the number of parent-reported barriers. Both lower level of family financial resourcing and having a non-binary child predicted parents’ rating of the extent of barriers. Child age and level of speech were predictors for reports of experiencing a higher number of facilitators, with parents of younger children or of non-speaking autistic children reporting a greater number of facilitators. We discuss how these results may be useful to support service delivery and identify areas for future research.

Research into the efficacy of support services for autistic children and their families suggests that a range of diverse support services are likely to have beneficial effects on expressive language, motor skills, emotional regulation, anxiety, daily living skills, and/or social functioning (Duncan et al., 2018; Hyman et al., 2020; Perihan et al., 2019; Ruggeri et al., 2020; Sandbank et al., 2021; Sissons et al., 2022; Tiede & Walton, 2019; Weiss et al., 2018). Unfortunately, many families with an autistic child report experiencing barriers that prevent or restrict their ability to access and/or participate in support services (Fong et al., 2022; Pickard & Ingersoll, 2016; Shepherd et al., 2018).

The types of barriers reported by parents/caregivers (herein referred to as parents) of autistic children include fractured service systems, lengthy wait lists, knowledge limitations, and/or financial pressures along with societal stigma and/or limited diversity in support services (Fong et al., 2022; Luelmo et al., 2020; Pickard & Ingersoll, 2016; Stahmer et al., 2019). Barriers experienced by New Zealand parents of autistic children appear to be similar to those reported globally, including financial, geographic, time, and service system barriers (Birkin et al., 2008; Stahmer et al., 2019). Though such barriers may also be faced by parents of children with other developmental differences, parents of autistic children comparatively appear to exhibit higher levels of parenting stress and/or decreased satisfaction with aspects of received support services (Amireh, 2019; Bitterman et al., 2008; Hayes & Watson, 2013; Picardi et al., 2018).

Parents also report factors that facilitate their access and participation in autism support services (referred to herein as facilitators), including easily accessible information and education on autism and/or support services, trusting relationships with providers, assistance with navigating service systems, peer support, and/or transparency of support service cost (Birkin et al., 2008; Lee & Meadan, 2021; Pearson & Meadan, 2018; Stahmer et al., 2019; Wilson et al., 2021). Currently, there does not appear to be any research that has quantified parent reported facilitators to autism support services.

Barriers and facilitators to support services are likely not experienced to the same degree among all families who have an autistic child/ren. Pickard and Ingersoll (2016), for example, found that parents with lower incomes reported less knowledge of autism support services, which in turn was associated with receiving a reduced number of such services. Several studies have found that lower income families access and participate in support services at a reduced rate (Carr et al., 2016; Nguyen et al., 2016; Smith et al., 2020). These data suggest there are likely additional challenges to accessing support services experienced by lower income families.

This study was conducted within Aotearoa, New Zealand. New Zealand has a multicultural population of over 5 million people (Statistics New Zealand, 2023). The most common ethnicities include European (70.2%), Māori (the indigenous peoples of New Zealand; 16.5%), Asian (15.1%), and Pacific (8.1%). The majority of the population speak English (95.4%), followed by te reo Māori (4%) and Samoan (2.2%; Statistics New Zealand, 2020a). Rates of child poverty in New Zealand pose numerous challenges, including issues of inequity. Approximately 16.3% or 187,300 New Zealand children are deemed to live in poverty (Gage et al., 2022; Statistics New Zealand, 2022). In addition, disabled children are slightly more likely to live in poverty compared to non-disabled children (Statistics New Zealand, 2022).

Autism diagnostic and support services are available in New Zealand through the government-funded public system or the user-pays private sector. The majority of autistic children in New Zealand (72%) gain a diagnosis through the public system with the process on average taking 10.9 months (van der Meer & Evans, 2021). Post-diagnosis, autism support services are scattered across multiple government agencies (Htut et al., 2019). While parents can elect to pay for private support services, this can be prohibitively expensive (Shepherd et al., 2018). Based on two recent New Zealand surveys, less than a quarter of parents reported satisfaction with autism support services (Eggleston et al., 2019; van der Meer & Evans, 2021).

Presently, there does not appear to be research which has explored the number and range of barriers or facilitators parents face, the degree to which those barriers/facilitators are experienced, nor the potential influence of certain sociodemographic variables, such as family income levels. An understanding of the factors that positively and negatively impact support service access and participation could be useful for developing more inclusive and effective support services for autistic children and their families.

Towards this end, the present study involved a quantitative survey to examine the types, number, and extent of barriers and facilitators to accessing and participating in support services as reported by New Zealand parents of autistic children. Principles of equity and the importance of equal access to services informed the development of the study questions. The concept of access, within this study, is not limited to the availability of services. Based on previous research examining healthcare equity, factors such as affordability, quality, and availability of information are also included (Goddard & Smith, 2001; Gulliford et al., 2002; Oliver & Massialos, 2004). The aims of this study were to (a) quantify the total number and extent of barriers and facilitators to support services as reported generally by New Zealand parents of an autistic child/ren, and (b) to identify if there were any significant differences based upon sociodemographic factors in general and family level of financial resourcing specifically due to the relatively high rates of income inequality and child poverty within New Zealand (OECD, 2022).

## Method

### Ethics and informed consent

Ethical approval was obtained from Victoria University of Wellington Human Ethics Committee (#29469). Participation in the study was voluntary and anonymous. Parents were provided with information relevant to the study and provided informed consent prior to starting the survey. Parents were asked not to include identifiable information on themselves or their child. Where identifying information was provided, it was removed.

### Participants

Screening questions were used at the start of the survey to ensure respondents currently resided in New Zealand and were the parent or primary caregiver of a child who (a) was 18 years of age or younger, and (b) had a diagnosis of autism. At the end of the 2-month data collection period, 195 surveys had been submitted through Qualtrics. Surveys with incomplete demographic information and no further responses (*n* = 22) were removed, resulting in a total of 173 surveys for analysis. Of this total, 173 parents completed the demographic and barriers sections of the survey and 143 completed the full survey. Depending upon the normality of the data, the relevant statistical tests (i.e. t-test, Mann Whitney U, Chi square) were used to determine whether there were any significant differences between those who only completed the barriers section and those who completed the full survey. No significant differences were found on any demographic variable. Descriptive statistical analysis was conducted to examine demographic characteristics of parent participants (Table S1). Parents reported that their autistic children’s current ages ranged from 2 years 6 months through to 18 years 7 months. Child age at the time of diagnosis ranged from 1 year 10 months through to 18 years 6 months. For those children who had a diagnosis in addition to autism (83.24%), the median number of additional diagnoses was two, with a range of one to eight. The most common additional diagnosis was anxiety. Child demographic characteristics are presented in Table S2.

### Distribution

A website was constructed to assist with dissemination of the survey. Information provided on this website included an outline of the survey content, participant eligibility as listed in the previous section, the process of consent, contact details of the first and fourth authors, a brief profile introducing the first author (e.g. information about her role and research interests), and embedded links to the online survey hosted by Qualtrics. The survey was distributed mainly online via email and/or Facebook pages through health and autism-specific organisations (e.g. Autism New Zealand, New Zealand Paediatrics Society, Wellington Early Intervention Trust). To ensure those who did not have reliable Internet access could participate, paper copies of the survey were available and distributed by mail to providers and potential participants upon request. Twenty paper copies were mailed out in equal amounts to two New Zealand–based providers. The questionnaire was open for responses between 22 September and 22 November 2021. Electronic responses were downloaded from the Qualtrics database onto a secure password protected USB drive. No paper-based surveys were returned.

### Materials

#### Survey development

The survey used in this study was based on the results of a qualitative systematic literature review (Wallace-Watkin et al., 2023). This review, conducted collectively by the authors, examined parent reported barriers and facilitators for accessing and/or participating in support services for autistic children from underserved families. Main themes identified within this review included support service accessibility, diversity of support services, and community (for subthemes see Table S3). Using the themes identified within the review as a guide, a list of brief barrier and facilitator statements was created. Upon completion of these written statements, the Barriers to Treatment Participation Scale (BTPS; Colonna-Pydyn et al., 2007; Kazdin et al., 1997) was reviewed to determine whether any relevant factors remained which had not yet been included in the survey. This resulted in the addition of a statement regarding parent/child illness as a barrier to support services. In total, 76 statements, split evenly between the barrier and facilitator sections, were placed into tables (see Table S3), and configured with Likert-type rating scales. Statements were then removed from thematic categories and randomly listed to create the final version of the survey.

#### Survey content

The questionnaire was divided into four sections: (a) informed consent and screening questions, (b) parent and child demographic information, (c) barriers to accessing and participating in professional support services, and (d) facilitators for accessing and participating in professional support services. The survey ended with an optional opportunity to list current support services and number of service hours received. The first section included four screening questions designed to check eligibility. In addition to the requirements that the child be 18 years of age or younger and diagnosed with autism, the respondent was asked to confirm that (a) they were one of the child’s parents/primary caregivers, and (b) the family currently resided in New Zealand.

Questions relevant to parent demographic information included the respondent’s relationship to their child, the number and type (e.g. child’s siblings, father) of individuals within the household, whether the respondent was the one who usually co-ordinated their child’s support services, ethnicity, location (i.e. urban/suburban or rural), highest education level held by respondent and/or by any individual within the household, and family level of financial resourcing. The term ‘lower income’ as used in this study refers to families who currently had, or were eligible for, a community services card^1^ and/or income tested benefit. Child demographic information included current age and age at diagnosis, additional diagnoses, gender, ethnicity, and expressive language. Parents who had more than one autistic child were asked to fill in the survey with one child in mind and then repeat the process for any additional children if they wished.

In the ‘barriers’ section, respondents were asked to rate potential barriers to any support service which they wanted to access, had accessed, and/or had participated in over the previous 12 months. Each possible rating (i.e. ‘not applicable’, ‘not at all a barrier’, ‘somewhat a barrier’, ‘a barrier’, and ‘very much a barrier’) was defined (see Table S4) before the list of 38 statements were provided in table format. Participants could then optionally add up to two additional barriers that had not been listed and/or provide further qualitative information regarding barriers.

In the ‘facilitators’ (listed as ‘helpers’) section, respondents were asked to rate helpful factors experienced within any support service which they wished to, or had, accessed and/or participated in during the previous 12 months. Each possible rating on the Likert-type scale (i.e. ‘I did not experience this’, ‘not helpful’, ‘somewhat helpful’, ‘helpful’ and ‘very helpful’) was defined (see Table S4) before the list of 38 statements were presented. Participants could then optionally add up to two additional facilitators that had not been listed and/or provide further qualitative information regarding facilitators. Finally, parents could choose to list any support services their child currently received, including number of hours per week those services were received.

### Data management and analysis

Quantitative analyses were conducted using IBM SPSS Statistics (Version 28.0.1) and *R* (Version 4.2.0). Descriptive statistics were calculated for both parent and child demographic information. Participants were able to select more than one ethnicity. For statistical analysis (e.g. regression models), participants were grouped into one of three groups: (1) European/Pākehā, (2) Māori, or (3) other ethnic group/s (i.e. those who were not Pākehā or Māori). A participant was categorised as ‘Māori’ if one of their stated ethnicities was Māori.

Cronbach’s alpha was calculated for the barrier/facilitator scales. The total number of barriers/facilitators experienced by participants was calculated, and the resulting dataset checked for normality through visual inspection of QQ plots and calculation of Kolmogorov-Smirnov tests. The mean total number of barriers/facilitators experienced, both overall and by participant level of financial resourcing (i.e. lower versus higher income) were calculated. As data were normally distributed, to locate any significant differences in total number of barriers/facilitators between lower and higher income participants, *t*-tests were conducted.

To calculate an extent score for barrier/facilitator statements, numeric values were assigned to the Likert-type scale responses (See Table S4). For each participant, an overall extent score was calculated for both barriers and facilitators. To ensure all values were represented in these extent scores, the mean Likert-type score was calculated across all statements. Co-occurrence of barriers and/or facilitators was examined through analysing percentage overlap. Statements were paired and the percentage of participants selecting both statements within a pair was calculated.

Responses to barrier/facilitator statements were grouped into original subthemes. The total number and percentage of participants who selected a barrier/facilitator statement within each subtheme was calculated. A score for extent of barriers/facilitators per subtheme was calculated as the mean Likert-type rating per participant across statements within each subtheme. Results are reported both overall and by level of financial resourcing (i.e. lower income versus higher income based on eligibility for a Community Services Card and/or income tested benefit). Prior to determining if any significant differences were present between barrier/facilitator subthemes, extent data were checked for normality. As the data were non-parametric, Friedman’s test and pairwise comparisons, corrected for multiple comparisons, were calculated. Thematic categories were ranked based on the results, with median and interquartile ranges reported.

Simultaneous multiple linear regressions were used to examine the predictors of (a) the total number and (b) the extent severity/helpfulness of parent-reported barriers and facilitators. Prior to conducting analyses, the normality of the datasets (i.e. the mean total number and extent of barriers/facilitators) was checked through visual inspection of QQ plots and calculation of Kolmogorov-Smirnov tests. As only one measure of financial resourcing was required, two variables (parent education level, relationship status) were excluded as these have been used as proxies and/or have previously been shown to be related to, socio-economic status (Durkin et al., 2017; Härkönen, 2018; Pickard & Ingersoll, 2016; Statistics New Zealand, 2020b). The age at which a child had been diagnosed as autistic was also excluded as this variable was highly correlated with the child’s current age. All excluded variables have been reported descriptively in Tables S1 and S2. The final set of included parent variables were (a) ethnicity, (b) level of financial resourcing (i.e. lower income versus higher income), (c) household location, and (d) number of child’s siblings. Child characteristics included (a) current child age, (b) presence versus absence of additional diagnoses, (c) gender and (d) language. A check for influential cases was conducted for all four resultant regression models using Cook’s distance, standardised DFBeta, and leverage values (Cook, 1977). Plots of residual values versus predicted values were also analysed ensuring the constant variance assumption was met in all models.

Additional barriers/facilitators noted by parents were collated into a table and reviewed by the first author. Where these aligned with a pre-defined statement within the main body of the survey, they were added to the count and rating of the most appropriate statement. Where new barriers and/or facilitators were identified, these were again collated, organised into the relevant theme, and re-written into barrier/facilitator statements. Totals and mean ratings were then calculated for each. The fourth author performed a check of 20% of the additional barriers and facilitators, with 92.6% agreement on the assigned theme. Disagreements were resolved via consensus. Due to there being too few responses to the questions regarding currently received support services and total hours of services received, these two variables were not included in the data analysis.

#### Community involvement

Feedback on the initial version of the survey was sought from autistic adults, parents of autistic children, community providers, a university-based statistician, and experienced researchers in the field of autism including a Māori post-doctoral researcher. Feedback was generally positive, with suggestions for improvements mainly regarding terminology. The majority of suggestions were incorporated into the survey. In the write-up of this study, preferences in terms of terminology have followed the feedback and guidance provided by autistic adults in Aotearoa, New Zealand (Monk, 2022).

## Results

### Barriers

#### Overall total and extent

A total of 173 parents selected and rated the barriers, they had experienced when accessing and/or participating in support services for their autistic child. Cronbach’s alpha across all barriers was 0.91, indicating strong internal consistency across scale items. The mean total number of barriers experienced across participants was 17.54 (*SD* = 7.74). There was a statistically significant difference between lower income and higher income parents on the total number of barriers experienced (*t* (169) = 3.17, *p* < 0.001). Lower income parents experienced a significantly higher total number of barriers (*M* = 19.52, *SD* = 7.72) than parents who were higher income (*M* = 15.85, *SD* = 7.39).

Results of Friedman’s test indicated that the extent to which participants experienced individual barriers was significantly different between statements (*X*^2^(37) = 1495.572, *p* < .001). Post hoc analysis using pairwise comparisons with Bonferroni correction for multiple tests revealed statistically significant differences between subthemes. Based on median scores per statement, lengthy waiting lists were experienced to a significantly greater extent than the majority of other barriers to support services (all *p* < .01; except B27 and B28 both *p* > .05). There were no significant differences between barrier statements based on family level of financial resourcing.

The extent to which participants experienced barriers was also significantly different across the different barrier subthemes (*X*^2^(8) = 484.011, *p* < .001). Post hoc analysis using pairwise comparisons revealed statistically significant differences in severity between subthemes (for full results see Table S5). Based on median scores per subtheme, Service pathway–related barriers were experienced to a significantly greater extent than culture, geographic location, social support, providers, service flexibility, and stigma-related barriers (all *p* < .05). Culture was experienced to a significantly lesser extent than all other barriers (all *p* < .05). There were no significant differences between subthemes based on family level of financial resourcing. Median extent scores, interquartile range, and the percentage of participants experiencing at least one barrier per subtheme are presented in Table 1.

**Table 1.** Median and interquartile range of the extent* to which each barrier theme/subtheme were experienced, rank of each subtheme, and percentage of participants selecting at least one barrier per theme/subtheme.

Barrier theme or subtheme	Group									Missing (n)
	All			Lower Income			Higher Income			
	*Mdn* (IQR)	Rank	%	*Mdn* (IQR)	Rank	%	*Mdn* (IQR)	Rank	%	
**Across all themes**	1.55 (0.48)	—	98.84	1.64 (0.59)	—	100	1.48 (0.44)	—	98.60	2
**Support service accessibility**	1.89 (0.61)	—	98.84	2 (0.68)	—	100.0	1.79 (0.63)	—	97.83	2
Knowledge and education	2.33 (1.33)	2	91.33	2.33 (1.33)	2	93.67	2.33 (1.33)	1	90.22	2
Service pathways	2.50 (1.25)	1	95.38	2.50 (1.25)	1	98.73	2.25 (1.0)	2	92.39	2
Financial pressure	2.0 (1.0)	3 =	86.71	2.0 (1.5)	3 =	88.61	2.0 (1.0)	3 =	85.87	2
Geographic location	1.40 (0.40)	8	79.77	1.6 (0.6)	8	84.81	1.4 (0.6)	7	73.91	2
Providers	1.60 (0.80)	5	80.12	1.8 (1.0)	5	86.08	1.4 (0.75)	8	75	2
**Diversity of support services**	1.50 (0.67)	—	89.60	1.58 (0.67)	—	93.67	1.38 (0.56)	—	86.96	2
Cultural and linguistic considerations	1.0 (0.17)	9	25.00	1.0 (0.33)	9	32.05	1.0 (0.0)	9	19.57	2
Service flexibility	1.56 (0.89)	6	89.02	1.67 (0.89)	6 =	92.41	1.5 (0.67)	5 =	86.96	2
**Community**	1.57 (0.71)	—	87.86	1.71 (1.0)	—	91.14	1.43 (0.57)	—	85.87	2
Stigma	2.0 (2.0)	3 =	64.16	2.0 (2.0)	3 =	73.42	2.0 (1.0)	3 =	57.61	2
Social support	1.50 (0.83)	7	83.24	1.67 (1.0)	6 =	86.08	1.5 (0.67)	5 =	83.70	2

IQR: interquartile range.

*Note.* *Extent is based on Numeric conversion of Likert-type scale ratings, where 1 = not at all a barrier/not applicable, 2 = somewhat a barrier, 3 = a barrier, 4 = very much a barrier.

#### Percentage overlap between statements

The percentage overlap between participants who selected each possible pair of barriers was calculated. The five most commonly selected co-occuring barriers were as follows: Not being sure what kinds of support services were available occurred alongside lengthy waiting lists (76%), not being sure which services were best for a child (76%), and not being able to find information on how to access support services (72%). Not being sure which support services were best for a child also occurred alongside length waiting lists (72%) and not being able to find information on how to access support services (67%). Full results of this analysis are available in Table S6.

#### Simultaneous multiple regressions

To evaluate what factors predicted parent reported total number and rating of barriers, two simultaneous multiple linear regressions were conducted. Data were approximately normally distributed, and all cases were retained. For the total number of barriers, the model was significant, *R*^2^ = 0.124, *F*(10,157) = 2.219, *p* = 0.019. This suggests that the model explained 12.4% of the variation in the total number of barriers. The results indicated no significant effects other than for family level of financial resourcing. Specifically, a lower family income was associated with a higher total number of barriers reported. For the overall mean rating across all barriers, the model was significant *R*^2^ = 0.112, *F*(10, 157) = 1.987, *p* = 0.038. This indicates that the model explained 11.2% of the variation in mean rating of the extent of barriers. The results indicated that lower income families and child gender (non-binary) were significantly associated with a higher mean rating of barriers reported (both *p* < 0.05). Full results are presented in Table 2.

**Table 2.** Simultaneous linear regression predicting total number and overall mean rating of barriers and facilitators.

Predictors	Overall total number of barriers		Overall mean rating across all barriers		Overall total number of facilitators		Overall mean rating across all facilitators	
	β	*p*	β	*p*	β	*p*	β	*p*
**Parent characteristics**								
Ethnicity								
Māori vs. European	0.105	0.188	0.029	0.720	−0.013	0.879	−0.046	0.610
Other vs. European	0.54	0.483	0.042	0.589	−0.002	0.979	0.011	0.899
Financial resourcing								
Lower income vs. Higher income	0.179	**0.023** *	0.169	**0.033** *	−0.107	0.210	−0.82	0.348
Household location								
Rural vs. non-rural	0.082	0.304	0.106	0.186	−0.019	0.826	−0.021	0.809
Child’s siblings								
Number of siblings	0.119	0.125	0.107	0.171	−0.065	0.443	−0.056	0.519
**Child characteristics**								
Current child age	−0.120	0.156	−0.001	0.988	−0.251	**0.007** **	−0.185	0.052
Presence vs. absence of additional diagnoses	0.114	0.162	0.80	0.330	0.072	0.422	0.077	0.402
Gender								
Female vs. Male/Non- binary	−0.025	0.748	0.022	0.779	−0.094	0.266	−0.087	0.312
Non-binary vs. Male/female	0.149	0.064	0.167	**0.040** *	−0.128	0.153	−0.101	0.272
Language								
Non-speaking vs. speaking	−0.084	0.295	−0.122	0.130	0.180	**0.043** *	0.176	0.053

*Note.* * ***p* ⩽ 0.05**, ** ***p* ⩽ 0.01**. Other ethnicity refers to parents who were from an underrepresented ethnic group other than Māori.

#### Other reported barriers

In total, 83 parents provided 118 responses (maximum of two per parent) to the optional question regarding additional experienced barriers. Of these responses, 19 were removed due to being: (a) a one-word or ambiguous response, (b) a general comment, (c) related to diagnosis, or (d) related to funding. A further 47 comments were responses which fit within one of the pre-existing barrier statements. The remaining 52 comments (from 43 parents) were collated and re-written into new barrier statements. This resulted in an additional 10 barrier statements which are presented, along with the mean rating of extent and total number of participants who had experienced each factor, within Table S7.

### Facilitators

#### Overall total and extent

A total of 143 parents (82.7%) selected and rated the facilitators they reported experiencing when accessing and/or participating in support services for their autistic child. Cronbach’s alpha across all facilitators was 0.95 indicating strong internal consistency across scale items. The mean total number of facilitators experienced across participants was 17.94 (*SD* = 9.46). Though lower income parents experienced slightly fewer facilitators (*M* = 16.54, *SD* = 9.19) compared to higher income parents (*M* = 19.05, *SD* = 9.58), this difference was not significant, *t*(140) = 1.58, *p* = 0.058.

The results of Friedman’s test indicated that the extent to which participants experienced individual facilitators was significantly different between statements (*X*^2^(37) = 867.176, *p* < 0.001). Post hoc analysis using pairwise comparisons with Bonferroni correction for multiple tests revealed statistically significant differences between subthemes. Based on median scores per statement, parent ability to advocate for their child was experienced as significantly most helpful compared to the majority of other facilitators to support services (all *p* < 0.01; except F26, F13, F6, F21, F25, F10, F9, F12, F11, and F2, all *p* > 0.05). There were no significant differences between facilitators statements based on family level of financial resourcing.

The extent to which participants found facilitators to be helpful was also significantly different across the different facilitator subthemes, *X*^2^(8) = 229.863, *p* < 0.001). Post hoc analysis with Bonferroni correction for multiple tests revealed statistically significant differences in helpfulness between subthemes (for full results see Table S5). Based on median scores per subtheme, provider-related facilitators were experienced as significantly more helpful than the majority of subthemes (all *p* < 0.001) except financial pressure (*p* > 0.05). Culture was significantly lower than service flexibility, stigma, geographic location, knowledge and education, financial pressure, and providers (all *p* < 0.05). There were no significant differences between subthemes based on family level of financial resourcing. Median helpfulness scores, interquartile range, and the percentage of participants experiencing at least one facilitator per subtheme are presented in Table 3.

**Table 3.** Median and interquartile range of the helpfulness* of each facilitator theme/subtheme, rank of each subtheme, and percentage of participants selecting at least one facilitator per theme/subtheme.

Facilitator group or subgroup	Group									Missing (n)
	All			Lower Income			Higher Income			
	*Mdn* (IQR)	Rank	%	*Mdn* (IQR)	Rank	%	*Mdn* (IQR)	Rank	%	
**Across all themes**	1.56 (0.59)	—	95.1	1.49 (0.56)	—	95.2	1.61 (0.72)	—	94.9	1
**Support service accessibility**	1.87 (0.65)	—	95.07	1.74 (0.69)	—	95.24	2.0 (1.74)	—	94.94	1
Knowledge and education	1.8 (0.8)	5	86.01	1.8 (0.8)	4	80.95	1.8 (1.0)	6	89.87	1
Service pathways	1.40 (0.6)	7	76.22	1.4 (0.4)	7	76.19	1.6 (1.0)	7	75.95	1
Financial pressure	2.0 (1.5)	2 =	82.52	2.0 (1.0)	1 =	82.54	2.5 (1.5)	1	83.54	1
Geographic location	1.83 (0.83)	4	88.11	1.67 (0.67)	5	87.30	2.0 (0.83)	3 =	88.61	1
Providers	2.4 (1.4)	1	88.81	2.0 (1.4)	1 =	87.30	2.4 (1.2)	2	89.87	1
**Diversity of support services**	1.64 (1.0)	—	86.01	1.64 (0.91)	—	82.54	1.64 (1.18)	—	88.61	1
Cultural and linguistic considerations	1 (0.67)	9	37.06	1.0 (1.0)	9	41.27	1.0 (0.33)	9	32.91	1
Service flexibility	1.75 (1.38)	6	85.31	1.63 (1.25)	6	82.54	1.88 (1.38)	5	87.34	1
**Community**	1.50 (1.0)	—	73.43	1.5 (1.0)	—	74.60	1.5 (1.25)	—	72.15	1
Stigma	2.0 (2.0)	2 =	53.85	2.0 (2.0)	1 =	53.97	2.0 (2.0)	3 =	53.16	1
Social support	1.33 (1.0)	8	56.64	1.33 (1.0)	8	57.14	1.33 (1.0)	8	56.96	1

IQR: interquartile range.

*Note.* *Helpfulness is based on Numeric conversion of Likert-type scale ratings, where 1 = not helpful/I did not experience this, 2 = somewhat helpful, 3 = helpful, 4 = very helpful.

#### Percentage overlap between statements

The percentage overlap between participants who selected each possible pair of facilitators was calculated. The five most commonly selected co-occuring facilitators were as follows: Easy communication with providers during sessions co-occured frequently with providers responding to parent calls and texts (69%), having a good relationship with the provider (68%), parent ability to advocate for their child (66%). The provider responding to parent calls and texts also frequently co-occurred alongside having a good relationship with the provider (65%). The fifth rated frequently co-occuring facilitators was tied between three statements. This included parent ability to advodcate for their child being experienced alongside having a good relationship with providers (64%) and providers responding to calls and texts (64%); and ease of communication with the providers occuring alongside the provider understanding the child and/or family’s needs (64%). Full results are available in Table S8.

#### Simultaneous multiple regressions

To evaluate what factors predicted parent-reported total number and rating of facilitators, two simultaneous multiple linear regressions were conducted, the results of which are presented in Table 2. The model predicting the mean rating of facilitators was skewed to the right. The mean rating data for this model only were log-transformed. The linear regression model predicting total number of facilitators had three high leverage values. Removal of these values had a limited impact on the model; therefore, results reported here are inclusive of these values.

The regression model for total number of facilitators was significant, *R*^2^ = 0.138, *F*(10, 128) = 2.047, *p* = 0.034. This indicates that the model explained 13.8% of the variance in total number of facilitators selected. Results indicated that current child age (the younger a child was) and a child’s level of expressive communication (non-speaking) were significantly associated with parents selecting a higher total number of facilitators. Transformed data were used in the conduction of the simultaneous regression for overall mean rating of facilitators. The resulting model was not significant, *R*^2^ = 0.095, *F*(10, 128) = 1.337, *p* = 0.218. No predictors significantly affect the mean rating of facilitators (see Table 2).

#### Other reported facilitators

A total of 14 parents listed additional facilitators resulting in 19 comments. Thirteen of these comments were excluded due to being one-word responses or unrelated to support services. A further two comments fit within one of the pre-existing facilitator statements. This resulted in four facilitator statements, which are presented along with the mean rating and total number of participants who had experienced each factor, in Table S9.

### Barriers and facilitators

#### Percentage overlap between statements

The percentage overlap between participants who selected each possible pair of barriers and facilitators was calculated. The five most commonly selected co-occuring barriers and facilitators were located, with lengthy waiting lists found to be the barrier in each pair. Lengthy waiting lists commonly co-occured with the faciltative factors of easy communication with providers during sessions (69%), parent ability to advocate for their child (69%), affordable or free support services (68%), the provider responding to parent calls, texts, and emails (68%), and having a good relationship with the provider (67%). Full results of this analysis are available in Table S10.

## Discussion

This sample of New Zealand parents reported several barriers and facilitators to autism support services with waiting lists reported as the greatest barrier and parental ability to advocate the greatest facilitator. Barriers related to the theme of service pathways were experienced to a significantly greater extent, whereas facilitators related to the theme of providers were rated as significantly most helpful. Lower income families reported significantly more barriers and reportedly experienced those barriers to a significantly greater extent than higher income families. Parents of non-binary gendered children experienced barriers to a significantly greater extent than parents of children who did not identify as non-binary. Parents of younger children, and of non-speaking children, reported a significantly higher number of facilitators; however, there were no significant differences in the mean rating (i.e. the rating of *how helpful* each factor was) based on any sociodemographic factor.

Almost all parents experienced barriers to autism support services. These barriers were selected from across a range of areas aligning with recent research, which suggests parents experience multiple differing barriers to accessing and participating in support services for their autistic child (Fong et al., 2022; Wallace-Watkin et al., 2023). Previous research results suggest that parents of autistic children exhibit higher rates of stress, report limited coordination of autism services, and/or have lower rates of satisfaction with aspects of those services (Amireh, 2019; Bitterman et al., 2008; Eggleston et al., 2019; Hayes & Watson, 2013). The results of this study provide further evidence that parents of autistic children face multiple challenges in this area.

The present study appears to be the first to compare the extent to which differing barriers impact parents, with waiting lists indicated to be the single greatest barrier. Wait lists have been identified as a barrier to support services across several studies (Birkin et al., 2008; Lim et al., 2021; Sun et al., 2013). While shorter wait times for support services have been associated with improved adaptive behaviour skills in young autistic children (Aishworiya et al., 2021), lengthy wait lists have been associated with higher levels of parental stress and/or lower ratings of parental emotional wellbeing (Rivard et al., 2014; Tait et al., 2016). Anecdotally, waiting lists for services, particularly government-funded services, may be up to 1 year (Connor, 2021). However, there does not appear to be New Zealand based research clarifying the average length of waiting lists generally or by service type. This would be an obvious area for future research.

Lower family income predicted a greater number and larger negative impact of reported barriers. A recent literature review conducted by Smith et al. (2020) examining autism support service disparities found that lower income families accessed services at a lower rate, experienced greater challenges in gaining referrals, and reported receiving a lower quality of care compared to families from higher socioeconomic backgrounds. The findings of the present study, specifically those which indicate that lower income families experience significantly more barriers, and experienced those barriers to a greater extent, may partially explain the causes behind service use disparities. Overall, service pathway barriers such as difficulty gaining referrals and lengthy waiting lists tended to be experienced by parents to a significantly greater extent. This indicates families need greater support in navigating service systems.

In the present study, parents of non-binary autistic children reported experiencing barriers to a greater extent. Though research suggests autistic individuals are more likely to identify as non-binary or gender diverse (Cooper et al., 2018; Corbett et al., 2023) very little is known regarding the experiences of this group when accessing and/or participating in support services. Carlile (2019) examined the experiences of gender diverse children and their families when accessing healthcare and found that the challenges faced by this group, including limited provider knowledge of gender diversity and long waiting lists, were experienced to a greater degree when the child was also autistic. Further research is needed to explore the experiences of gender diverse autistic youth when accessing autism services.

Most parents experienced at least one facilitator to support services. Overall, facilitators which related to the theme providers such as having a good relationship with a provider, easy communication, and providers understanding the family’s and child’s needs, were rated as most helpful. The reported facilitators are consistent with other reports which have highlighted the importance of certain provider traits, such as showing respect, having a willingness to advocate on behalf of families, and prioritising warm relationships (Birkin et al., 2008; Chlebowski et al., 2018; Stahmer et al., 2019).

The single most helpful factor reported by parents was the ability to advocate for their child. Learning to advocate for an autistic child is likely to be a process which necessitates parents increase their personal knowledge of autism and learn advocacy skills (Blanche et al., 2015; Pearson & Meadan, 2018; Smith-Young et al., 2022). Within qualitative research, parents have reported seeking out information, both online and from other parents of autistic children to advocate for their autistic child (Lee & Meadan, 2021; Pearson & Meadan, 2018; Stahmer et al., 2019). Given that service pathway barriers were found to be experienced to a significantly greater extent than other barriers, the effect of imparting knowledge and education which empowers parents to navigate such systems and advocate for their child is an important area for future research.

Within the present study, significant predictors of experiencing a greater number of facilitators included having a younger child or a child who was non-speaking. One potential reason for the difference between age groups may be the present focus on early support (Noyes-Grosser et al., 2018; Rojas-Torres et al., 2020). In addition, younger children may have higher communication needs, with speech and language services highly utilised within New Zealand (Kasilingam et al., 2021).

The five most commonly co-occuring barrier/facilitator pairs all included lengthy waiting lists as the barrier. The greatest overlap was between lengthy waiting lists as a barrier, and the helpfulness of parental advocacy. Previous research has also found that parents utilise advocacy skills to overcome barriers faced when accessing services (Smith-Young et al., 2022). An important issue is whether effective advocacy could in fact help to overcome the barrier of long waiting lists.

The study aimed to recruit parents from a wide range of differing sociodemographic backgrounds. According to results of the most recent census, the two largest ethnic groups within New Zealand are Pākehā (New Zealand European/European) and Māori (Statistics New Zealand, 2020a). The number of Māori whānau who participated (17.9%) was representative of the New Zealand population (16.5%; Statistics New Zealand, 2020a). The same was true for the number of Pākehā participants, with 72.8% of survey participants selecting this ethnicity compared to the general population estimate for this group of 70.2% (Statistics New Zealand, 2020a). Therefore, the recommendations discussed are likely to be fairly representative of the two largest ethnic groups in New Zealand.

### Implications

The results of this study suggest the value of increasing parent advocacy skills, increasing knowledge of autism in parents and the public, and reducing waiting times. Ensuring all providers have the skills, tools, personnel, and knowledge to appropriately support families with autistic children would assist families in gaining and participating in support services for their autistic child. Dissemination of strengths-based information regarding autism and support services could also be beneficial. Additional supports likely need to be put in place to assist lower income families given the findings of the present study which indicate such families experience a comparatively greater number and extent of barriers, and comparatively fewer facilitators. By doing so, we may begin to address the current inequality faced by families when accessing and participating in autism support services. It is also essential that appropriate levels of funding are provided to meet these goals.

### Limitations and future directions

As only a small number of participants provided information regarding the support services they were receiving, it was not possible to explore connections between barriers/facilitators and specific services. Though the number of participants from Pākehā and Māori backgrounds were representative of the New Zealand population, the number of participants from other cultural groups, such as Pacific peoples, was much lower than the New Zealand population estimates (Statistics New Zealand, 2020a). Future research specifically exploring barriers and facilitators for differing ethnic and/or cultural groups is strongly recommended. It is important to understand the experiences of New Zealand parents who have little or no knowledge of the English language. Due to funding limitations, the survey used in this study was only available in English; therefore, the results may not be representative of New Zealand parents who primarily speak other languages. Although the number of Māori participants was representative of the New Zealand population in general, future research which follows Kaupapa Māori^2^ principles which is offered in Te Reo Māori would be beneficial in future explorations of barriers/facilitators specifically for Māori.

Due to the survey being based on self-report, the potential for recall bias also needs to be considered. Finally, though paper-based surveys were made available, and a range of agencies were contacted, all responses were provided online. There is potential that participants without Internet access may have had less opportunity to learn of, and therefore participate in, the study.

Future research could seek to explore the potential reasons parents of older autistic children experience fewer facilitative factors to support services, how to reduce long waitlists, and how to enable lower income families to access and participate in services that meet their needs and those of their autistic child. Examining the ways in which families with an autistic child may be supported while on waiting lists for support services would also be a useful direction for future research. Finally, New Zealand based research which explores how barriers and facilitators may intersect and influence service use would be useful. A qualitative approach may be helpful in further understanding how families may be utilising facilitators to overcome barriers to autism support services.

## Conclusion

New Zealand parents reported several barriers and facilitators to accessing and participating in autism support services. Differences based on sociodemographic factors, with a specific focus on level of family financial resourcing, were identified. Results suggest steps need to be taken to address the number of barriers parents face, increase facilitative factors, and improve equitable access to, and participation in, support services for autistic children.

## Supplemental Material

sj-docx-1-aut-10.1177_13623613231168240 – Supplemental material for Parent reported barriers and facilitators to support services for autistic children in Aotearoa New Zealand Click here for additional data file. Supplemental material, sj-docx-1-aut-10.1177_13623613231168240 for Parent reported barriers and facilitators to support services for autistic children in Aotearoa New Zealand by Carla Wallace-Watkin, Jeff Sigafoos, Lisa Woods and Hannah Waddington in Autism

## Notes

1. A community services card (CSC) assists families with the costs of healthcare in New Zealand. This initiative is income-based and targets lower income New Zealand families/whānau (Ministry of Health, 2022)

2. Kaupapa Māori, in reference to research, refers to the embeding of Māori values, practices, and beliefs as the centre of the research process. There is emphasis on the collective sharing of knowledge (Rolleston et al., 2022).

## References

[bibr1-13623613231168240] Aishworiya R. Goh T. J. Sung M. Tay S. K. H. (2021). Correlates of adaptive skills in children with autism spectrum disorder. Autism, 25(6), 1592–1600. 10.1177/1362361321997287 33726526

[bibr2-13623613231168240] Amireh M. M. H. (2019). Stress levels and coping strategies among parents of children with autism and Down syndrome: The effect of demographic variables on levels of stress. Child Care in Practice, 25(2), 146–156. 10.1080/13575279.2018.1446907

[bibr3-13623613231168240] Birkin C. Anderson A. Seymour F. Moore D. W. (2008). A parent-focused early intervention program for autism: Who gets access? Journal of Intellectual & Developmental Disability, 33(2), 108–116. 10.1080/13668250802036746 18569398

[bibr4-13623613231168240] Bitterman A. Daley T. C. Misra S. Carlson E. Markowitz J. (2008). A national sample of pre-schoolers with autism spectrum disorders: Special education services and parent satisfaction. Journal of Autism and Developmental Disorders, 38(8), 1509–1517. 10.1007/s10803-007-0531-9 18228122

[bibr5-13623613231168240] Blanche E. I. Diaz J. Barretto T. Cermak S. A. (2015). Caregiving experiences of Latino families with children with autism spectrum disorder. The American Journal of Occupational Therapy, 69(5), 6905185010. 10.5014/ajot.2015.017848 26356658

[bibr6-13623613231168240] Carlile A. (2019). The experiences of transgender and non-binary children and young people and their parents in healthcare settings in England, UK: Interviews with members of a family support group. International Journal of Transgender Health, 21(1), 16–32. 10.1080/15532739.2019.1693472 33015656 PMC7430470

[bibr7-13623613231168240] Carr T. Shih W. Lawton K. Lord C. King B. Kasari C. (2016). The relationship between treatment attendance, adherence, and outcome in a caregiver-mediated intervention for low-resourced families of young children with autism spectrum disorder. Autism, 20(6), 643–652. 10.1177/1362361315598634 26290524

[bibr8-13623613231168240] Chlebowski C. Magaña S. Wright B. Brookman-Frazee L. (2018). Implementing an intervention to address challenging behaviours for autism spectrum disorder in publicly funded mental health services: Therapist and parent perceptions of delivery with Latinx families. Cultural Diversity and Ethnic Minority Psychology, 24(4), 552–563. 10.1037/cdp0000215 30024185 PMC6188834

[bibr9-13623613231168240] Colonna-Pydyn C. Gjesfjeld C. D. Greeno C. G. (2007). The factor structure of the Barriers to Treatment Participation Scale (BTPS): Implications for future barriers scale development. Administration and Policy in Mental Health and Mental Health Services Research, 34(6), 563–569. 10.1007/s10488-007-0139-6 17943436

[bibr10-13623613231168240] Connor F. (2021, April 13). ‘An absolute joke’: Autistic boy left on waitlist for over a year despite early diagnosis. https://www.newshub.co.nz/home/new-zealand/2021/05/an-absolute-joke-autistic-boy-left-on-waitlist-for-over-a-year-despite-early-diagnosis.html

[bibr11-13623613231168240] Cook R. D. (1977). Detection of influential observation in linear regression. Technometrics, 19(1), 15–18. 10.2307/1268249

[bibr12-13623613231168240] Cooper K. Smith L. G. E. Russell A. J. (2018). Gender identity in autism: Sex differences in social affiliation with gender groups. Journal of Autism and Developmental Disorders, 48(12), 3995–4006. 10.1007/s10803-018-3590-1 29705922 PMC6223803

[bibr13-13623613231168240] Corbett B. A. Muscatello R. A. Klemencic M. E. West M. Kim A. Strang J. F. (2023). Greater gender diversity among autistic children by self-report and parent-report. Autism, 27(1), 158–172. 10.1177/13623613221085337 35363085 PMC9525458

[bibr14-13623613231168240] Duncan A. Ruble L. A. Meinzen-Derr J. Thomas C. Stark L. J. (2018). Preliminary efficacy of a daily living skills intervention for adolescents with high-functioning autism spectrum disorder. Autism, 22(8), 983–994. 10.1177/1362361317716606 28914086 PMC7370244

[bibr15-13623613231168240] Durkin M. S. Maenner M. J. Baio J. Christensen D. Daniels J. Fitzgerald R. Imm P. Lee L.-C. Schieve L. A. Van Naarden Braun K. Wingate M. S. Yeargin-Allsopp M. (2017). Autism spectrum disorder among US children (2002–2010): Socioeconomic, racial, and ethnic disparities. American Journal of Public Health, 107(11), 1818–1826. 10.2105/AJPH.2017.304032 28933930 PMC5637670

[bibr16-13623613231168240] Eggleston M. J. F. Thabrew H. Frampton C. M. A. Eggleston K. H. F. Hennig S. C. (2019). Obtaining an autism spectrum disorder diagnosis and supports: New Zealand parents’ experiences. Research in Autism Spectrum Disorders, 62, 18–25. 10.1016/j.rasd.2019.02.004

[bibr17-13623613231168240] Fong V. C. Lee B. S. Iarocci G. (2022). A community-engaged approach to examining barriers and facilitators to accessing autism services in Korean immigrant families. Autism, 26(2), 525–537. 10.1177/13623613211034067 34286622 PMC8814949

[bibr18-13623613231168240] Gage R. Chambers T. Smith M. McKerchar C. Puloka V. Pearson A. Kawachi I. Signal L. (2022). Children’s perspectives on the problem of child poverty in Aotearoa New Zealand: A wearable camera study. New Zealand Medical Journal, 135(1559), 95–111. 10.26635/6965.5420 35999785

[bibr19-13623613231168240] Goddard M. Smith P. (2001). Equity of access to health care services: Theory and evidence from the UK. Social Science & Medicine, 53, 1149–1162. 10.1016/S0277-9536(00)00415-9 11556606

[bibr20-13623613231168240] Gulliford M. Figueroa-Munoz J. Morgan M. Hughes D. Gibson B. Beech R. Hudson M. (2002). What does ‘access to health care’ mean? Journal of Health Services Research & Policy, 7(3), 186–188. 10.1258/13558190276008 12171751

[bibr21-13623613231168240] Härkönen J. (2018). Single-mother poverty: How much do educational differences in single motherhood matter? In Nieuwenhuis R. Maldonado L. C. (Eds.), The triple bind of single-parent families: Resources, employment and policies to improve wellbeing (pp. 31-50). Policy Press.

[bibr22-13623613231168240] Hayes S. A. Watson S. L. (2013). The impact of parenting stress: A meta-analysis of studies comparing the experience of parenting stress in parents of children with and without autism spectrum disorder. Journal of Autism and Developmental Disorders, 43(3), 629–642. 10.1007/s10803-012-1604-y 22790429

[bibr23-13623613231168240] Htut M. Ho E. Wiles J. (2019). A study of Asian children who are diagnosed with autism spectrum disorder and available support services in Auckland, New Zealand. Journal of Autism and Developmental Disorders, 50, 1855–1865. 10.1007/s10803-019-03936-y 30820725

[bibr24-13623613231168240] Hyman S. L. Levy S. E. Myers S. M ., & Council on Children with Disabilities, Section on Developmental and Behavioral Pediatrics. (2020). Identification, evaluation, and management of children with autism spectrum disorder. Pediatrics, 145(1), e20193447. 10.1542/peds.2019-3447 31843864

[bibr25-13623613231168240] Kasilingam N. Waddington H. van der Meer L. (2021). Early intervention for children with autism spectrum disorder in New Zealand: What children get and what parents want. International Journal of Disability, Development and Education, 68(4), 521–537. 10.1080/1034912X.2019.1696949

[bibr26-13623613231168240] Kazdin A. E. Holland L. Crowley M. Breton S. (1997). Barriers to Treatment Participation Scale: Evaluation and validation in the context of child outpatient treatment. Journal of Child Psychology and Psychiatry, 38(8), 1051–1062. 10.1111/j.1469-7610.1997.tb01621.x 9413802

[bibr27-13623613231168240] Lee J. D. Meadan H. (2021). Children with autism spectrum disorders in low-resource settings: Reported experiences and needs of parents in Mongolia. Journal of Autism and Developmental Disorders, 51(10), 3586–3599. 10.1007/s10803-020-04818-4 33387240

[bibr28-13623613231168240] Lim N. O’Reilly M. Sigafoos J. Lancioni G. E. Sanchez N. J. (2021). A review of barriers experienced by immigrant parents of children with autism when accessing services. Review Journal of Autism and Developmental Disorders, 8(3), 366–372. 10.1007/s40489-020-00216-9

[bibr29-13623613231168240] Luelmo P. Sandoval Y. Kasari C. (2020). Undocumented Mexican mothers of children with autism: Navigating the health care and educational service systems. International Journal of Developmental Disabilities, 68, 1–11. 10.1080/20473869.2020.1850159 PMC9351561 35937177

[bibr30-13623613231168240] Ministry of Health. (2022, 30 June). Community services card. https://www.health.govt.nz/our-work/primary-health-care/primary-health-care-subsidies-and-services/community-services-card

[bibr31-13623613231168240] Monk R. (2022). Autism terminology guidance from the Autistic community of Aotearoa New Zealand: A living resource created by Autistic people with the support of Autism New Zealand. Autism New Zealand. https://autismnz.org.nz/resources/autism-new-zealand-terminology-guide/

[bibr32-13623613231168240] Nguyen C. T. Krakowiak P. Hansen R. Hertz-Picciotto I. Angkustsiri K. (2016). Sociodemographic disparities in intervention service utilization in families of children with autism spectrum disorder. Journal of Autism and Developmental Disorders, 46(12), 3729–3738. 10.1007/s10803-016-2913-3 27639855 PMC5112120

[bibr33-13623613231168240] Noyes-Grosser D. M. Elbaum B. Wu Y. Siegenthaler K. M. Cavalari R. S. Gillis J. M. Romanczyk R. G. (2018). Early intervention outcomes for toddlers with autism spectrum disorder and their families. Infants & Young Children, 31(3), 177–199. 10.1097/IYC.0000000000000121

[bibr34-13623613231168240] OECD. (2022). OECD economic surveys; New Zealand. https://www.oecd.org/economy/surveys/New%20Zealand-2022-OECD-economic-survey-overview.pdf

[bibr35-13623613231168240] Oliver A. Massialos E. (2004). Equity of access to health care: Outlining the foundations for action. Journal of Epidemiological Community Health, 58, 655–658. 10.1136/jech.2003.017731 PMC1732867 15252067

[bibr36-13623613231168240] Pearson J. N. Meadan H. (2018). African American parents’ perceptions of diagnosis and services for children with autism. Education and Training in Autism and Developmental Disabilities, 53(1), 17–32.

[bibr37-13623613231168240] Perihan C. Burke M. Bowman-Perrot L. Bicer A. Gallup J. Thompson J. Sallese M. (2019). Effects of cognitive behavioural therapy for reducing anxiety in children with high functioning ASD: A systematic review and meta-analysis. Journal of Autism and Developmental Disorders, 50, 1958–1972. 10.1007/s10803-019-03949-7 30810842

[bibr38-13623613231168240] Picardi A. Gigantesco A. Tarolla E. Stoppioni V. Cerbo R. Cremonte M. Alessandri G. Lega I. Nardocci F. (2018). Parental burden and its correlates in families of children with autism spectrum disorder: A multicentre study with two comparison groups. Clinical Practice & Epidemiology in Mental Health, 14(1), 143–176. 10.2174/1745017901814010143 30158998 PMC6080067

[bibr39-13623613231168240] Pickard K. E. Ingersoll B. R. (2016). Quality versus quantity: The role of socioeconomic status on parent-reported service knowledge, service use, unmet service needs, and barriers to service use. Autism, 20(1), 106–115. 10.1177/1362361315569745 25948601

[bibr40-13623613231168240] Rivard M. Terroux A. Parent-Boursier C. Mercier C. (2014). Determinants of stress in parents of children with autism spectrum disorders. Journal of Autism and Developmental Disorders, 44(7), 1609–1620. 10.1007/s10803-013-2028-z 24384673

[bibr41-13623613231168240] Rojas-Torres L. P. Alonso-Esteban Y. Alcantud-Marín F. (2020). Early intervention with parents of children with autism spectrum disorders: A review of programs. Children, 7(12), 294. 10.3390/children7120294 33333900 PMC7765314

[bibr42-13623613231168240] Rolleston A. Miskelly P. McDonald M. Wiles J. Poppe K. Doughty R. (2022). Cultural context in New Zealand: Incorporating Kaupapa Māori values in clinical research and practice. Health Promotion International, 37(3), 1–12. 10.1093/heapro/daac065 35788305

[bibr43-13623613231168240] Ruggeri A. Dancel A. Johnson R. Sargent B. (2020). The effect of motor and physical activity intervention on motor outcomes of children with autism spectrum disorder: A systematic review. Autism, 24(3), 544–568. 10.1177/1362361319885215 31782658

[bibr44-13623613231168240] Sandbank M. Bottema-Beutel K. Woynaroski T. (2021). Intervention recommendations for children with autism in light of a changing evidence base. Journal of the American Medical Association: Pediatrics, 175(4), 341–342. 10.1001/jamapediatrics.2020.4730 33165523

[bibr45-13623613231168240] Shepherd D. Csako R. Landon J. Goedeke S. Ty K. (2018). Documenting and understanding parent’s intervention choices for their child with autism spectrum disorder. Journal of Autism and Developmental Disorders, 48(4), 988–1001. 10.1007/s10803-017-3395-7 29214603

[bibr46-13623613231168240] Sissons J. H. Blakemore E. Shafi H. Skotny N. Lloyd D. M. (2022). Calm with horses? A systematic review of animal-assisted interventions for improving social functioning in children with autism. Autism, 26(6), 1320–1340. 10.1177/13623613221085338 35403450 PMC9344573

[bibr47-13623613231168240] Smith K. A. Gehricke J.-G. Iadarola S. Wolfe A. Kuhlthau K. A. (2020). Disparities in service use among children with autism: A systematic review. Pediatrics, 145(Suppl. 1), S35–S46. 10.1542/peds.2019-1895G 32238530

[bibr48-13623613231168240] Smith-Young J. Chafe R. Audas R. Gustafson D. L. (2022). ‘I know how to advocate’: Parents’ experiences in advocating for children and youth diagnosed with autism spectrum disorder. Health Services Insights, 15, 11786329221078803. 10.1177/11786329221078803 PMC8883377 35237049

[bibr49-13623613231168240] Stahmer A. C. Vejnoska S. Iadarola S. Straiton D. Segovia F. R. Luelmo P. Morgan E. H. Lee H. S. Javed A. Bronstein B. Hochheimer S. Cho E. Aranbarri A. Mandell D. Hassrick E. M. Smith T. Kasari C. (2019). Caregiver voices: Cross-cultural input on improving access to autism services. Journal of Racial and Ethnic Health Disparities, 6(4), 752–773. 10.1007/s40615-019-00575-y 30859514 PMC6936957

[bibr50-13623613231168240] Statistics New Zealand. (2020a, September 3). Ethnic group summaries reveal New Zealand’s multicultural make-up. https://www.stats.govt.nz/news/ethnic-group-summaries-reveal-new-zealands-multicultural-make-up/

[bibr51-13623613231168240] Statistics New Zealand. (2020b, September 14). Wellbeing outcomes worse for sole parents. https://www.stats.govt.nz/news/wellbeing-outcomes-worse-for-sole-parents/

[bibr52-13623613231168240] Statistics New Zealand. (2022, February 24). Child poverty statistics: Year ended June 2021. https://www.stats.govt.nz/information-releases/child-poverty-statistics-year-ended-june-2021/

[bibr53-13623613231168240] Statistics New Zealand. (2023, January 27). Population. https://www.stats.govt.nz/topics/population

[bibr54-13623613231168240] Sun X. Allison C. Auyeung B. Matthews F. E. Baron-Cohen S. Brayne C. (2013). Service provision for autism in mainland China: Preliminary mapping of service pathways. Social Science & Medicine, 98, 87–94. 10.1016/j.socscimed.2013.08.016 24331886 PMC6345370

[bibr55-13623613231168240] Tait K. Fung F. Hu A. Sweller N. Wang W. (2016). Understanding Hong Kong Chinese families’ experiences of an autism/ASD diagnosis. Journal of Autism and Developmental Disorders, 46(4), 1164–1183. 10.1007/s10803-015-2650-z 26572657

[bibr56-13623613231168240] Tiede G. Walton K. M. (2019). Meta-analysis of naturalistic developmental behavioral interventions for young children with autism spectrum disorder. Autism, 23(8), 2080–2095. 10.1177/1362361319836371 31018655

[bibr57-13623613231168240] van der Meer L. Evans K . (2021). The autism diagnostic process in New Zealand: Final Report. Autism CRC.

[bibr58-13623613231168240] Wallace-Watkin C. Sigafoos J. Waddington H. (2023). Barriers and facilitators for obtaining support services among underserved families with an autistic child: A systematic qualitative review. Autism, 27(3), 588–601. 10.1177/13623613221123712 36081366

[bibr59-13623613231168240] Weiss J. A. Thomson K. Burnham Riosa P. Albaum C. Chan V. Maughan A. Tablon P. Black K. (2018). A randomized waitlist-controlled trial of cognitive behavior therapy to improve emotion regulation in children with autism. Journal of Child Psychology and Psychiatry, 59(11), 1180–1191. 10.1111/jcpp.12915 29687457 PMC6220772

[bibr60-13623613231168240] Wilson M. Whelan T. Milne L. Hamilton D. Jacobs D. Pilkington P. (2021). A thematic analysis of influences on parents’ autism intervention decisions. Research in Developmental Disabilities, 117, 104035. 10.1016/j.ridd.2021.104035 34329855

