# Supplementary material for: Parent reported barriers and facilitators to support services for autistic children in Aotearoa New Zealand
Source: Autism. 2023 Apr 27;27(8):2542–54. doi: 10.1177/13623613231168240 (PMC10576898; doi:10.1177/13623613231168240)
Supplement: sj-docx-1-aut-10.1177_13623613231168240 – Supplemental material for Parent reported barriers and facilitators to support services for autistic children in Aotearoa New Zealand [file sj-docx-1-aut-10.1177_13623613231168240.docx]

**Supplementary Material**

**Table S1**

*Demographic Characteristics of Parents by Response Type*

| **Demographic characteristic** | Barriers | | |  | Facilitators | | |
| --- | --- | --- | --- | --- | --- | --- | --- |
|  | Mean (SD) | *n* | % |  | Mean (SD) | *n* | % |
| Relationship to child |  |  |  |  |  |  |  |
| Mother |  | 163 | 94.2 |  |  | 136 | 95.1 |
| Father |  | 4 | 2.3 |  |  | 3 | 2.1 |
| Other (grandmother; birthing parent) |  | 4 | 2.3 |  |  | 3 | 1.4 |
| Preferred not to say |  | 2 | 1.2 |  |  | 1 | 0.7 |
| Parent relationship |  |  |  |  |  |  |  |
| Single parent |  | 40 | 23.1 |  |  | 31 | 21.7 |
| Two parents |  | 132 | 76.3 |  |  | 112 | 78.3 |
| Preferred not to say |  | 1 | 0.6 |  |  | - | - |
| Child’s sibling status |  |  |  |  |  |  |  |
| No siblings |  | 48 | 27.7 |  |  | 43 | 30.1 |
| At least one sibling | 1.3 (1.2) | 124 | 71.7 |  | 1.2 (1.2) | 100 | 69.9 |
| Preferred not to say |  | 1 | 0.6 |  |  | - | - |
| Parent ethnicity |  |  |  |  |  |  |  |
| New Zealand European/European |  | 126 | 72.8 |  |  | 105 | 73.4 |
| Māori |  | 31 | 17.9 |  |  | 25 | 17.5 |
| Other |  | 15 | 8.7 |  |  | 12 | 8.4 |
| Preferred not to say |  | 1 | 0.6 |  |  | 1 | 0.7 |
| Household location |  |  |  |  |  |  |  |
| Urban/Suburban |  | 152 | 87.9 |  |  | 128 | 89.5 |
| Rural |  | 20 | 11.6 |  |  | 15 | 10.5 |
| Preferred not to say |  | 1 | 0.6 |  |  | - | - |
| Household highest level of education |  |  |  |  |  |  |  |
| Trade or secondary school qualification |  | 54 | 31.2 |  |  | 43 | 30.1 |
| University degree |  | 115 | 66.5 |  |  | 99 | 69.2 |
| Preferred not to say |  | 4 | 2.3 |  |  | 1 | 0.7 |
| Household level of resourcing |  |  |  |  |  |  |  |
| Lower Income |  | 78 | 45.6 |  |  | 63 | 44.1 |
| Higher Income |  | 93 | 54.4 |  |  | 79 | 55.2 |
| Preferred not to say |  | 2 | 1.2 |  |  | 1 | 0.7 |

**Table S2**

*Demographic Characteristics for the Children of Parent Participants*

| **Demographic characteristic** | Barriers | | |  | Facilitators | | |
| --- | --- | --- | --- | --- | --- | --- | --- |
|  | Mean (SD) | *n* | % |  | Mean (SD) | *n* | % |
| Child age |  |  |  |  |  |  |  |
| Current (Years.Months) | 11.1 (3.7) |  |  |  | 11.3 (4) |  |  |
| At diagnosis (Years.Months) | 6.7 (3.7) |  |  |  | 6.8 (3.8) |  |  |
| Additional diagnoses |  |  |  |  |  |  |  |
| No additional diagnoses |  | 29 | 16.8 |  |  | 21 | 14.7 |
| One or more additional diagnoses | 1.8 (1.5) | 144 | 83.2 |  | 1.9 (1.6) | 122 | 85.3 |
| *Types of additional diagnoses |  |  |  |  |  |  |  |
| Anxiety |  | 76 | 43.9 |  |  | 66 | 46.2 |
| ADHD/ADD |  | 72 | 41.6 |  |  | 63 | 44.1 |
| Global development delay |  | 24 | 13.9 |  |  | 17 | 11.9 |
| Depression |  | 20 | 11.6 |  |  | 16 | 11.2 |
| Intellectual disability |  | 22 | 12.7 |  |  | 17 | 11.9 |
| Dyspraxia |  | 15 | 8.7 |  |  | 14 | 9.8 |
| Sensory processing difficulties |  | 11 | 6.4 |  |  | 10 | 7.0 |
| Specific learning disability (e.g., dyslexia,  dysgraphia) |  | 9 | 5.2 |  |  | 9 | 6.3 |
| Auditory processing disorder |  | 6 | 3.5 |  |  | 5 | 3.5 |
| Other |  | 55 | 31.8 |  |  | 51 | 35.7 |
| Child gender |  |  |  |  |  |  |  |
| Male |  | 129 | 74.6 |  |  | 106 | 74.1 |
| Female |  | 35 | 20.2 |  |  | 29 | 20.3 |
| Non-binary |  | 7 | 4.0 |  |  | 7 | 4.9 |
| Preferred not to say |  | 2 | 1.2 |  |  | 1 | 0.7 |
| Child ethnicity |  |  |  |  |  |  |  |
| New Zealand European/European |  | 113 | 65.3 |  |  | 94 | 66.7 |
| Māori |  | 38 | 22.0 |  |  | 30 | 21.0 |
| Other |  | 20 | 11.6 |  |  | 17 | 11.9 |
| Preferred not to say |  | 2 | 1.2 |  |  | 2 | 1.4 |
| Level of language |  |  |  |  |  |  |  |
| Speaking |  | 160 | 92.5 |  |  | 135 | 94.4 |
| Non-speaking |  | 11 | 6.4 |  |  | 7 | 4.9 |
| Preferred not to say |  | 2 | 1.2 |  |  | 1 | 0.7 |

*Note.* * Parents were able to select more than one additional diagnosis, therefore the totals sum to more than 173 (100%)

**Table S3**

*Barrier and Facilitator Statements for Each Thematic Grouping*

| Theme or subtheme | Statement |
| --- | --- |
| **Support service accessibility** |  |
| Knowledge and education | Barriers   1. I could not find information on how to access support service(s) (B8) 2. I’m not sure which support service(s) are best for my child (B27) 3. I’m not sure what kinds of support service(s) are available (B28)   Facilitators   1. I knew how to find information about available support service(s) (F27) 2. The support service helped me to better understand my child and their needs (F21) 3. I was able to easily find information on support service(s) which would suit my child (F30) 4. I was able to easily find information about how to access a support service(s) (F8) 5. I was able to advocate for my child (F28) |
| Service pathways | Barriers   1. I had difficulty gaining a referral for the support service(s) (B14) 2. I could not find a clinician or provider who was able/willing to help me access support service(s) (B7) 3. My child was not the right age for the support service(s) (B17) 4. Waiting lists for the support service(s) were too long (B18)   Facilitators   1. A professional quickly referred me to a support service(s) (F15) 2. I had help/support in accessing the support service(s) (F7) 3. My child was seen soon after referral (F17) 4. Accessing the support service(s) was a simple process (F29) 5. I was provided with information on “next steps” for my child when the support service(s) ended (F36) |
| Financial pressure | Barriers   1. The support service(s) cost too much (B2) 2. The time of sessions did not suit my work schedule or a family member’s work schedule (B3)   Facilitators   1. The support service(s) was affordable or free (F2) 2. I and/or a family member were able to work from home or take time off work so my child could access the support service(s) (F3) |
| Geographic location | Barriers   1. There was nobody providing the support service(s) in my local area (B13) 2. I had no access to transport (B25) 3. There was no affordable/accessible parking at the support service(s) (B26) 4. The support service(s) was delivered in my home (B37) 5. The support service(s) was delivered in a clinic or community setting (B38)   Facilitators   1. There were a range of support services in my local area (F14) 2. I had access to transport to get to and from sessions (F25) 3. There was plenty of affordable/accessible parking at the support service(s) (F26) 4. The support service(s) was delivered in my home (F37) 5. The support service(s) was delivered in a clinic or community setting (F38) 6. I was able to easily find information on local provider(s) (F31) |
| Providers | Barriers   1. I did not have a good relationship with the provider (B9) 2. The provider did not appear to understand my child’s/family’s needs (B10) 3. The provider did not respond to my phone calls/emails/texts (B11) 4. The provider requested too much personal information (B12) 5. It was difficult to communicate with the provider during the support service session(s) (B15)   Facilitators   1. I had a good relationship with the provider (F9) 2. The provider understood my child’s and/or family’s needs (F10) 3. The provider responded to my calls/texts/emails (F12) 4. The provider did not ask for too much personal information (F13) 5. It was easy to communicate with the provider during support service sessions (F11) |
| **Diversity of support services** |  |
| Cultural and linguistic  considerations | Barriers   1. The support service(s) were only offered in English (B1) 2. The support service(s) conflicted with my cultural beliefs (B4) 3. The support service(s) were not responsive to my/my child’s cultural needs/values (B5)   Facilitators   1. The support service(s) was offered in more than one language (F1) 2. The support service(s) approaches were responsive to my/my child’s cultural beliefs (F5) 3. My and/or my child’s culture was acknowledged in the support service (F4) |
| Service flexibility | Barriers   1. There were not enough opportunities for me to provide input into the support service(s) (B35) 2. The available appointment times were not suitable (B16) 3. My child refused to attend the support service(s) (B23) 4. The support service(s) sessions were too long (B29) 5. The support service(s) was not individualised to my child and/or my family (B30) 6. The support service did not align with my parenting approach (B31) 7. I was given too much homework from the support service sessions (B33) 8. The strategies taught in the support service(s) were too confusing or difficult to learn (B34) 9. The support service(s) was of low quality (B36)   Facilitators   1. The support service(s) had a range of times to choose from when scheduling an appointment (F16) 2. It was easy to reschedule appointments when needed (F24) 3. The support service(s) was individualised to my child and family (F32) 4. The support service(s) taught me helpful strategies to work with my child (F33) 5. The length of support service sessions was flexible and adapted to my family’s needs (F34) 6. My input and feedback were used in the support service(s) (F35) 7. My child enjoyed the support service(s) (F19) 8. The support service(s) was a good fit with my parenting approach (F6) |
| **Community** |  |
| Stigma | Barriers   1. I felt my parenting would be/was judged (B6)   Facilitators   1. My child’s strengths were highlighted and shared with me by the provider (F20) |
| Social support | Barriers   1. A family member disagreed with the support service(s) (B22) 2. I had obligations to extended family/friends which needed to be prioritised (B24) 3. Taking part in the support service added/would add stress (B32) 4. Stressful personal event(s) made attending the support service(s) difficult (B21) 5. My child and/or a family member’s illness made attending the support service(s) difficult (B20) 6. I was unable to access childcare during the support service(s) (B19)   Facilitators   1. Other adult family members (e.g. other parent, grandparent, aunt or uncle) were included in the support service(s) (F22) 2. My child’s siblings were included in the support service(s) (F23) 3. I had access to reliable childcare (F18) |

**Table S4**

*Definitions Provided for Each Possible Response to Barrier and Facilitator Statements*

| Possible Response | Numeric Value | Definition Provided |
| --- | --- | --- |
| Barriers |  |  |
| Not applicable | 1 | This is not applicable to me/my child |
| Not at all a barrier | 1 | This is something you have not experienced or, if you have, it was not a barrier |
| Somewhat a barrier | 2 | This was a challenge which was relatively easily solved |
| A barrier | 3 | This was a definite challenge which was not easily solved |
| Very much a barrier | 4 | This was very much a challenge. It could not be solved and/or I could not access or participate in the support service because of this. |
| Facilitators |  |  |
| I did not experience this | 1 | You have not experienced the helper stated. |
| Not helpful | 1 | Mark this box if you have experienced the helper stated but it was not helpful. |
| Somewhat helpful | 2 | This was only a little helpful, it did not make a large difference. |
| Helpful | 3 | This was helpful to the support service(s) for my child |
| Very helpful | 4 | This made a big difference and/or was vital to the support service(s) for my child/family |

**Table S5**

*Median and p-value Results of Posthoc Pairwise Comparison Tests of Significance Between Barrier and Facilitator Subthemes*

| Subtheme | Facilitator  Median | Knowledge & education | Service pathways | Financial pressure | Geographic location | Providers | Culture | Service flexibility | Stigma | Social support | Barrier Median |
| --- | --- | --- | --- | --- | --- | --- | --- | --- | --- | --- | --- |
| Knowledge & education | 1.8 | - | 1 | .093 | **<.001** | **<.001** | **<.001** | **<.001** | **<.001** | **<.001** | 2.3 |
| Service pathways | 1.4 | **.005** | - | .072 | **<.001** | **<.001** | **<.001** | **<.001** | **<.001** | **<.001** | 2.5 |
| Financial pressure | 2.0 | .070 | **<.001** | - | **<.001** | **<.001** | **<.001** | **<.001** | .90 | **<.001** | 2.0 |
| Geographic location | 1.8 | 1 | .010 | **.034** | - | **.023** | **.003** | **<.001** | **<.001** | **.035** | 1.4 |
| Providers | 2.4 | **<.001** | **<.001** | 1 | **<.001** | - | **<.001** | 1 | **.034** | 1 | 1.6 |
| Culture | 1.0 | **<.001** | .25 | **<.001** | **<.001** | **<.001** | - | **<.001** | **<.001** | **<.001** | 1 |
| Service flexibility | 1.75 | 1 | **.032** | **.011** | 1 | **<.001** | **<.001** | - | .76 | 1 | 1.6 |
| Stigma | 2.0 | 1 | **.019** | **.019** | 1 | **<.001** | **<.001** | 1 | - | .**022** | 2.0 |
| Social support | 1.3 | **<.001** | 1 | **<.001** | **.001** | **<.001** | 1.0 | **.002** | **.001** | - | 1.5 |

*Note.* Significance values have been adjusted by Bonferroni correction for multiple tests

**Bold values** indicate a significant difference

| Barriers | Facilitators |
| --- | --- |

**Table S6**

*Percentage of Participants Selecting Both Paired Barrier Statements*

| Barrier Number | Percent | | | | | | | | | | | | | | | | | | | | | | | | | | | | | | | | | | | | |
| --- | --- | --- | --- | --- | --- | --- | --- | --- | --- | --- | --- | --- | --- | --- | --- | --- | --- | --- | --- | --- | --- | --- | --- | --- | --- | --- | --- | --- | --- | --- | --- | --- | --- | --- | --- | --- | --- |
|  | **B1** | **B2** | **B3** | **B4** | **B5** | **B6** | **B7** | **B8** | **B9** | **B**  **10** | **B**  **11** | **B**  **12** | **B**  **13** | **B**  **14** | **B**  **15** | **B**  **16** | **B**  **17** | **B**  **18** | **B**  **19** | **B**  **20** | **B**  **21** | **B**  **22** | **B**  **23** | **B**  **24** | **B**  **25** | **B**  **26** | **B**  **27** | **B**  **28** | **B**  **29** | **B**  **30** | **B**  **31** | **B**  **32** | **B**  **33** | **B**  **34** | **B**  **35** | **B**  **36** | **B**  **37** |
| **B1** |  |  |  |  |  |  |  |  |  |  |  |  |  |  |  |  |  |  |  |  |  |  |  |  |  |  |  |  |  |  |  |  |  |  |  |  |  |
| **B2** | 4 |  |  |  |  |  |  |  |  |  |  |  |  |  |  |  |  |  |  |  |  |  |  |  |  |  |  |  |  |  |  |  |  |  |  |  |  |
| **B3** | 5 | 51 |  |  |  |  |  |  |  |  |  |  |  |  |  |  |  |  |  |  |  |  |  |  |  |  |  |  |  |  |  |  |  |  |  |  |  |
| **B4** | 2 | 10 | 9 |  |  |  |  |  |  |  |  |  |  |  |  |  |  |  |  |  |  |  |  |  |  |  |  |  |  |  |  |  |  |  |  |  |  |
| **B5** | 2 | 21 | 17 | 10 |  |  |  |  |  |  |  |  |  |  |  |  |  |  |  |  |  |  |  |  |  |  |  |  |  |  |  |  |  |  |  |  |  |
| **B6** | 4 | 48 | 47 | 10 | 19 |  |  |  |  |  |  |  |  |  |  |  |  |  |  |  |  |  |  |  |  |  |  |  |  |  |  |  |  |  |  |  |  |
| **B7** | 4 | 52 | 49 | 9 | 19 | 49 |  |  |  |  |  |  |  |  |  |  |  |  |  |  |  |  |  |  |  |  |  |  |  |  |  |  |  |  |  |  |  |
| **B8** | 4 | 55 | 54 | 8 | 17 | 52 | **60** |  |  |  |  |  |  |  |  |  |  |  |  |  |  |  |  |  |  |  |  |  |  |  |  |  |  |  |  |  |  |
| **B9** | 2 | 31 | 32 | 10 | 14 | 33 | 36 | 35 |  |  |  |  |  |  |  |  |  |  |  |  |  |  |  |  |  |  |  |  |  |  |  |  |  |  |  |  |  |
| **B10** | 3 | 48 | 49 | 10 | 20 | 50 | 55 | 55 | 39 |  |  |  |  |  |  |  |  |  |  |  |  |  |  |  |  |  |  |  |  |  |  |  |  |  |  |  |  |
| **B11** | 1 | 34 | 34 | 5 | 12 | 36 | 38 | 38 | 27 | 37 |  |  |  |  |  |  |  |  |  |  |  |  |  |  |  |  |  |  |  |  |  |  |  |  |  |  |  |
| **B12** | 1 | 17 | 20 | 5 | 7 | 21 | 19 | 20 | 16 | 21 | 16 |  |  |  |  |  |  |  |  |  |  |  |  |  |  |  |  |  |  |  |  |  |  |  |  |  |  |
| **B13** | 2 | 43 | 45 | 8 | 15 | 43 | 49 | 54 | 31 | 46 | 35 | 18 |  |  |  |  |  |  |  |  |  |  |  |  |  |  |  |  |  |  |  |  |  |  |  |  |  |
| **B14** | 2 | 52 | 51 | 8 | 19 | 50 | 57 | **60** | 36 | 52 | 38 | 20 | 47 |  |  |  |  |  |  |  |  |  |  |  |  |  |  |  |  |  |  |  |  |  |  |  |  |
| **B15** | 3 | 31 | 34 | 7 | 15 | 36 | 38 | 37 | 29 | 38 | 27 | 16 | 31 | 36 |  |  |  |  |  |  |  |  |  |  |  |  |  |  |  |  |  |  |  |  |  |  |  |
| **B16** | 4 | 35 | 49 | 7 | 11 | 36 | 38 | 39 | 24 | 37 | 27 | 17 | 34 | 39 | 26 |  |  |  |  |  |  |  |  |  |  |  |  |  |  |  |  |  |  |  |  |  |  |
| **B17** | 3 | 33 | 36 | 4 | 11 | 33 | 34 | 37 | 20 | 32 | 27 | 15 | 33 | 35 | 24 | 29 |  |  |  |  |  |  |  |  |  |  |  |  |  |  |  |  |  |  |  |  |  |
| **B18** | 4 | **62** | **62** | 9 | 20 | 58 | **60** | **66** | 38 | 58 | 43 | 22 | 54 | **64** | 40 | 48 | 41 |  |  |  |  |  |  |  |  |  |  |  |  |  |  |  |  |  |  |  |  |
| **B19** | 3 | 26 | 30 | 4 | 6 | 24 | 24 | 27 | 16 | 25 | 19 | 11 | 24 | 28 | 16 | 25 | 21 | 34 |  |  |  |  |  |  |  |  |  |  |  |  |  |  |  |  |  |  |  |
| **B20** | 2 | 30 | 30 | 9 | 16 | 32 | 30 | 29 | 22 | 32 | 21 | 15 | 26 | 31 | 23 | 25 | 20 | 35 | 16 |  |  |  |  |  |  |  |  |  |  |  |  |  |  |  |  |  |  |
| **B21** | 3 | 41 | 40 | 9 | 17 | 43 | 39 | 41 | 27 | 39 | 30 | 18 | 34 | 38 | 31 | 32 | 27 | 49 | 20 | 30 |  |  |  |  |  |  |  |  |  |  |  |  |  |  |  |  |  |
| **B22** | 1 | 21 | 22 | 4 | 11 | 25 | 23 | 24 | 18 | 25 | 20 | 12 | 20 | 25 | 20 | 18 | 20 | 27 | 13 | 17 | 20 |  |  |  |  |  |  |  |  |  |  |  |  |  |  |  |  |
| **B23** | 1 | 37 | 38 | 8 | 15 | 36 | 34 | 38 | 23 | 34 | 27 | 17 | 32 | 35 | 26 | 28 | 25 | 43 | 20 | 24 | 32 | 20 |  |  |  |  |  |  |  |  |  |  |  |  |  |  |  |
| **B24** | 2 | 16 | 19 | 4 | 8 | 29 | 16 | 17 | 9 | 16 | 12 | 10 | 14 | 17 | 14 | 15 | 13 | 23 | 11 | 13 | 18 | 12 | 4 |  |  |  |  |  |  |  |  |  |  |  |  |  |  |
| **B25** | 2 | 9 | 9 | 1 | 4 | 9 | 8 | 8 | 4 | 8 | 5 | 4 | 5 | 7 | 5 | 8 | 6 | 10 | 4 | 5 | 9 | 4 | 5 | 5 |  |  |  |  |  |  |  |  |  |  |  |  |  |
| **B26** | 2 | 19 | 24 | 5 | 8 | 24 | 22 | 24 | 15 | 23 | 17 | 12 | 19 | 22 | 19 | 21 | 17 | 23 | 14 | 18 | 22 | 14 | 20 | 10 | 6 |  |  |  |  |  |  |  |  |  |  |  |  |
| **B27** | 3 | 56 | 56 | 8 | 17 | 53 | 56 | **67** | 35 | 55 | 38 | 22 | 52 | **60** | 36 | 43 | 35 | **71** | 29 | 29 | 42 | 23 | 41 | 18 | 9 | 23 |  |  |  |  |  |  |  |  |  |  |  |
| **B28** | 3 | **61** | **60** | 9 | 18 | 56 | **60** | **72** | 37 | 58 | 42 | 23 | 55 | **64** | 38 | 46 | 38 | **76** | 32 | 32 | 46 | 25 | 43 | 20 | 10 | 25 | **76** |  |  |  |  |  |  |  |  |  |  |
| **B29** | 2 | 22 | 24 | 6 | 9 | 25 | 21 | 23 | 17 | 23 | 17 | 11 | 22 | 23 | 18 | 23 | 16 | 27 | 17 | 19 | 24 | 14 | 18 | 11 | 4 | 12 | 25 | 26 |  |  |  |  |  |  |  |  |  |
| **B30** | 4 | 45 | 45 | 9 | 19 | 43 | 44 | 47 | 31 | 48 | 28 | 17 | 41 | 46 | 33 | 35 | 34 | 54 | 25 | 28 | 35 | 23 | 34 | 17 | 8 | 20 | 49 | 50 | 24 |  |  |  |  |  |  |  |  |
| **B31** | 2 | 36 | 34 | 9 | 15 | 40 | 36 | 36 | 27 | 38 | 23 | 17 | 30 | 35 | 27 | 26 | 24 | 44 | 21 | 26 | 33 | 20 | 27 | 15 | 5 | 16 | 37 | 38 | 20 | 36 |  |  |  |  |  |  |  |
| **B32** | 4 | 45 | 48 | 9 | 17 | 50 | 46 | 50 | 32 | 46 | 35 | 21 | 43 | 47 | 33 | 37 | 32 | 58 | 27 | 32 | 45 | 24 | 37 | 21 | 8 | 22 | 54 | 57 | 26 | 42 | 41 |  |  |  |  |  |  |
| **B33** | 2 | 28 | 30 | 6 | 11 | 31 | 27 | 29 | 20 | 29 | 22 | 14 | 26 | 28 | 22 | 26 | 22 | 34 | 19 | 20 | 28 | 18 | 22 | 15 | 3 | 15 | 30 | 33 | 21 | 27 | 27 | 35 |  |  |  |  |  |
| **B34** | 2 | 24 | 27 | 5 | 9 | 26 | 22 | 28 | 18 | 24 | 18 | 14 | 25 | 27 | 21 | 20 | 20 | 33 | 16 | 19 | 25 | 17 | 23 | 14 | 4 | 15 | 31 | 31 | 18 | 28 | 24 | 32 | 23 |  |  |  |  |
| **B35** | 3 | 34 | 34 | 8 | 15 | 35 | 34 | 38 | 28 | 34 | 27 | 15 | 32 | 40 | 30 | 27 | 25 | 42 | 20 | 24 | 29 | 20 | 25 | 16 | 5 | 18 | 38 | 40 | 19 | 35 | 33 | 36 | 24 | 24 |  |  |  |
| **B36** | 2 | 37 | 34 | 10 | 17 | 37 | 37 | 38 | 27 | 36 | 27 | 15 | 31 | 39 | 28 | 26 | 22 | 42 | 17 | 23 | 32 | 17 | 27 | 13 | 5 | 17 | 37 | 40 | 18 | 34 | 33 | 37 | 24 | 21 | 32 |  |  |
| **B37** | 2 | 8 | 9 | 2 | 4 | 10 | 8 | 7 | 6 | 8 | 6 | 6 | 6 | 8 | 7 | 8 | 6 | 10 | 6 | 7 | 9 | 6 | 7 | 7 | 3 | 5 | 8 | 9 | 5 | 8 | 8 | 11 | 7 | 5 | 8 | 8 |  |
| **B38** | 1 | 27 | 28 | 5 | 11 | 27 | 25 | 28 | 17 | 24 | 17 | 12 | 23 | 27 | 19 | 22 | 19 | 31 | 16 | 18 | 24 | 13 | 21 | 11 | 7 | 13 | 31 | 32 | 14 | 24 | 23 | 27 | 18 | 19 | 21 | 19 | 7 |

*Note.* **Bold values** indicate that 60% or more of participants selected both statements

**Table S7**

*Mean Rating and Number of Parents Selecting Each Additional Barrier*

| New Code | M (SD) | *n* |
| --- | --- | --- |
| 1. My child was declined support based on the support service/s  criteria | 3.87 (0.35) | 14 |
| 2. The provider appeared to have limited knowledge of autism or  autism-specific supports | 3.83 (0.39) | 13 |
| 3. The support service I would like my child to receive is not  currently offered. | 4 (0) | 5 |
| 4. I do not agree with the approaches taken within the support  service/s | 4 (0) | 3 |
| 5. The provider was unwilling to collaborate with other services | 3.33 (0.58) | 3 |
| 6. There was a lack of consistency in and/or between services | 3.33 (0.58) | 3 |
| 7. There was no follow-up from the provider | 4 (0) | 2 |
| 8. My child’s difficulties were focused on rather than their  strengths | 4 (0) | 2 |
| 9. There were long waiting times for meetings, planning, and/or  initiation of services once my child began the support service | 3 (0) | 1 |
| 10. The support service did not have multi-cultural providers | 3 (0) | 1 |

**Table S8**

*Percentage of Participants Selecting Both Paired Facilitator Statements*

| Facilitator  Number | Percent | | | | | | | | | | | | | | | | | | | | | | | | | | | | | | | | | | | | |
| --- | --- | --- | --- | --- | --- | --- | --- | --- | --- | --- | --- | --- | --- | --- | --- | --- | --- | --- | --- | --- | --- | --- | --- | --- | --- | --- | --- | --- | --- | --- | --- | --- | --- | --- | --- | --- | --- |
|  | **F**  **1** | **F2** | **F3** | **F4** | **F5** | **F6** | **F7** | **F8** | **F9** | **F**  **10** | **F**  **11** | **F**  **12** | **F**  **13** | **F**  **14** | **F**  **15** | **F**  **16** | **F**  **17** | **F**  **18** | **F**  **19** | **F**  **20** | **F**  **21** | **F**  **22** | **F**  **23** | **F**  **24** | **F**  **25** | **F**  **26** | **F**  **27** | **F**  **28** | **F**  **29** | **F**  **30** | **F**  **31** | **F**  **32** | **F**  **33** | **F**  **34** | **F**  **35** | **F**  **36** | **F**  **37** |
| **F1** |  |  |  |  |  |  |  |  |  |  |  |  |  |  |  |  |  |  |  |  |  |  |  |  |  |  |  |  |  |  |  |  |  |  |  |  |  |
| **F2** | 3 |  |  |  |  |  |  |  |  |  |  |  |  |  |  |  |  |  |  |  |  |  |  |  |  |  |  |  |  |  |  |  |  |  |  |  |  |
| **F3** | 1 | 38 |  |  |  |  |  |  |  |  |  |  |  |  |  |  |  |  |  |  |  |  |  |  |  |  |  |  |  |  |  |  |  |  |  |  |  |
| **F4** | 3 | 27 | 17 |  |  |  |  |  |  |  |  |  |  |  |  |  |  |  |  |  |  |  |  |  |  |  |  |  |  |  |  |  |  |  |  |  |  |
| **F5** | 3 | 24 | 15 | 27 |  |  |  |  |  |  |  |  |  |  |  |  |  |  |  |  |  |  |  |  |  |  |  |  |  |  |  |  |  |  |  |  |  |
| **F6** | 3 | 54 | 36 | 27 | 27 |  |  |  |  |  |  |  |  |  |  |  |  |  |  |  |  |  |  |  |  |  |  |  |  |  |  |  |  |  |  |  |  |
| **F7** | 1 | 36 | 24 | 19 | 17 | 33 |  |  |  |  |  |  |  |  |  |  |  |  |  |  |  |  |  |  |  |  |  |  |  |  |  |  |  |  |  |  |  |
| **F8** | 1 | 31 | 22 | 16 | 16 | 29 | 28 |  |  |  |  |  |  |  |  |  |  |  |  |  |  |  |  |  |  |  |  |  |  |  |  |  |  |  |  |  |  |
| **F9** | 3 | 57 | 38 | 27 | 28 | 57 | 38 | 34 |  |  |  |  |  |  |  |  |  |  |  |  |  |  |  |  |  |  |  |  |  |  |  |  |  |  |  |  |  |
| **F10** | 3 | 51 | 35 | 26 | 28 | 55 | 34 | 31 | **62** |  |  |  |  |  |  |  |  |  |  |  |  |  |  |  |  |  |  |  |  |  |  |  |  |  |  |  |  |
| **F11** | 3 | 59 | 41 | 28 | 27 | **60** | 38 | 33 | **68** | **64** |  |  |  |  |  |  |  |  |  |  |  |  |  |  |  |  |  |  |  |  |  |  |  |  |  |  |  |
| **F12** | 3 | 59 | 39 | 29 | 29 | 59 | 38 | 33 | **65** | **61** | **69** |  |  |  |  |  |  |  |  |  |  |  |  |  |  |  |  |  |  |  |  |  |  |  |  |  |  |
| **F13** | 4 | 49 | 28 | 23 | 25 | 46 | 31 | 28 | 51 | 48 | 52 | 55 |  |  |  |  |  |  |  |  |  |  |  |  |  |  |  |  |  |  |  |  |  |  |  |  |  |
| **F14** | 1 | 20 | 10 | 11 | 12 | 21 | 17 | 17 | 21 | 20 | 22 | 22 | 17 |  |  |  |  |  |  |  |  |  |  |  |  |  |  |  |  |  |  |  |  |  |  |  |  |
| **F15** | 1 | 36 | 20 | 15 | 17 | 34 | 28 | 24 | 36 | 36 | 40 | 39 | 31 | 17 |  |  |  |  |  |  |  |  |  |  |  |  |  |  |  |  |  |  |  |  |  |  |  |
| **F16** | 1 | 37 | 25 | 20 | 21 | 38 | 28 | 27 | 38 | 38 | 40 | 42 | 36 | 16 | 26 |  |  |  |  |  |  |  |  |  |  |  |  |  |  |  |  |  |  |  |  |  |  |
| **F17** | 0 | 24 | 17 | 13 | 13 | 24 | 19 | 15 | 28 | 26 | 27 | 26 | 24 | 13 | 19 | 20 |  |  |  |  |  |  |  |  |  |  |  |  |  |  |  |  |  |  |  |  |  |
| **F18** | 0 | 24 | 15 | 13 | 12 | 22 | 17 | 17 | 25 | 23 | 26 | 24 | 21 | 10 | 17 | 19 | 13 |  |  |  |  |  |  |  |  |  |  |  |  |  |  |  |  |  |  |  |  |
| **F19** | 2 | 41 | 26 | 22 | 23 | 44 | 26 | 25 | 45 | 42 | 46 | 45 | 34 | 18 | 26 | 31 | 21 | 18 |  |  |  |  |  |  |  |  |  |  |  |  |  |  |  |  |  |  |  |
| **F20** | 2 | 43 | 27 | 22 | 21 | 46 | 30 | 28 | 50 | 48 | 50 | 49 | 36 | 20 | 30 | 33 | 22 | 21 | 40 |  |  |  |  |  |  |  |  |  |  |  |  |  |  |  |  |  |  |
| **F21** | 3 | 52 | 36 | 24 | 25 | 56 | 36 | 30 | **62** | 59 | **62** | 58 | 45 | 20 | 36 | 38 | 27 | 24 | 45 | 50 |  |  |  |  |  |  |  |  |  |  |  |  |  |  |  |  |  |
| **F22** | 1 | 31 | 19 | 15 | 15 | 27 | 17 | 18 | 32 | 31 | 34 | 32 | 25 | 12 | 20 | 22 | 13 | 15 | 24 | 27 | 33 |  |  |  |  |  |  |  |  |  |  |  |  |  |  |  |  |
| **F23** | 1 | 14 | 8 | 6 | 8 | 14 | 13 | 10 | 17 | 17 | 18 | 18 | 15 | 10 | 12 | 12 | 10 | 10 | 13 | 14 | 17 | 13 |  |  |  |  |  |  |  |  |  |  |  |  |  |  |  |
| **F24** | 1 | 36 | 20 | 19 | 21 | 34 | 27 | 23 | 37 | 38 | 37 | 39 | 32 | 14 | 24 | 33 | 20 | 19 | 29 | 31 | 37 | 26 | 15 |  |  |  |  |  |  |  |  |  |  |  |  |  |  |
| **F25** | 2 | 43 | 29 | 18 | 17 | 38 | 29 | 25 | 45 | 40 | 45 | 45 | 35 | 17 | 27 | 28 | 18 | 19 | 31 | 32 | 43 | 27 | 14 | 26 |  |  |  |  |  |  |  |  |  |  |  |  |  |
| **F26** | 2 | 43 | 29 | 20 | 19 | 38 | 27 | 25 | 43 | 40 | 46 | 45 | 34 | 19 | 28 | 27 | 19 | 19 | 32 | 32 | 40 | 23 | 13 | 24 | 42 |  |  |  |  |  |  |  |  |  |  |  |  |
| **F27** | 1 | 34 | 23 | 17 | 17 | 33 | 25 | 29 | 36 | 35 | 38 | 38 | 30 | 17 | 27 | 27 | 19 | 18 | 29 | 31 | 35 | 17 | 12 | 27 | 27 | 29 |  |  |  |  |  |  |  |  |  |  |  |
| **F28** | 4 | 58 | 38 | 29 | 27 | **61** | 38 | 33 | **64** | 59 | **66** | **64** | 50 | 21 | 36 | 41 | 27 | 26 | 45 | 52 | **62** | 35 | 17 | 40 | 48 | 46 | 36 |  |  |  |  |  |  |  |  |  |  |
| **F29** | 1 | 25 | 17 | 15 | 16 | 26 | 22 | 22 | 28 | 27 | 31 | 31 | 26 | 15 | 21 | 21 | 18 | 15 | 22 | 23 | 26 | 14 | 10 | 20 | 20 | 23 | 24 | 29 |  |  |  |  |  |  |  |  |  |
| **F30** | 2 | 21 | 15 | 15 | 15 | 23 | 19 | 22 | 25 | 25 | 26 | 26 | 24 | 14 | 19 | 22 | 16 | 12 | 20 | 21 | 22 | 12 | 8 | 20 | 16 | 19 | 21 | 27 | 22 |  |  |  |  |  |  |  |  |
| **F31** | 1 | 27 | 20 | 11 | 13 | 24 | 22 | 24 | 28 | 25 | 28 | 29 | 24 | 14 | 20 | 23 | 14 | 13 | 22 | 23 | 23 | 13 | 9 | 20 | 19 | 22 | 24 | 26 | 20 | 20 |  |  |  |  |  |  |  |
| **F32** | 3 | 43 | 29 | 22 | 22 | 44 | 31 | 26 | 50 | 49 | 48 | 48 | 36 | 19 | 29 | 33 | 22 | 18 | 38 | 42 | 46 | 26 | 15 | 33 | 34 | 34 | 28 | 49 | 22 | 20 | 22 |  |  |  |  |  |  |
| **F33** | 3 | 48 | 33 | 24 | 23 | 50 | 30 | 28 | 51 | 48 | 52 | 50 | 38 | 20 | 32 | 35 | 21 | 24 | 41 | 44 | 53 | 27 | 16 | 35 | 38 | 37 | 33 | 55 | 22 | 21 | 24 | 41 |  |  |  |  |  |
| **F34** | 2 | 37 | 24 | 20 | 21 | 41 | 29 | 25 | 41 | 38 | 41 | 41 | 32 | 17 | 26 | 35 | 20 | 20 | 35 | 38 | 42 | 21 | 14 | 31 | 29 | 27 | 29 | 43 | 20 | 17 | 20 | 36 | 40 |  |  |  |  |
| **F35** | 2 | 43 | 29 | 22 | 22 | 45 | 31 | 28 | 48 | 45 | 48 | 49 | 37 | 20 | 29 | 35 | 20 | 20 | 38 | 41 | 48 | 29 | 16 | 33 | 36 | 33 | 31 | 52 | 25 | 22 | 24 | 41 | 41 | 37 |  |  |  |
| **F36** | 1 | 28 | 23 | 14 | 15 | 31 | 22 | 25 | 35 | 35 | 35 | 34 | 29 | 14 | 24 | 24 | 18 | 16 | 28 | 32 | 34 | 20 | 11 | 24 | 26 | 27 | 28 | 35 | 20 | 21 | 24 | 29 | 31 | 27 | 29 |  |  |
| **F37** | 2 | 39 | 24 | 18 | 20 | 37 | 29 | 23 | 40 | 40 | 42 | 41 | 33 | 16 | 28 | 30 | 20 | 20 | 30 | 34 | 41 | 23 | 16 | 32 | 27 | 26 | 26 | 42 | 22 | 19 | 19 | 32 | 37 | 31 | 34 | 24 |  |
| **F38** | 3 | 47 | 30 | 23 | 20 | 47 | 28 | 24 | 45 | 42 | 50 | 46 | 36 | 18 | 29 | 29 | 17 | 20 | 36 | 35 | 43 | 24 | 11 | 25 | 36 | 38 | 29 | 48 | 24 | 21 | 21 | 36 | 40 | 29 | 35 | 25 | 28 |

*Note.* **Bold values** indicate that 60% or more of participants selected both statements

**Table S9**

*Rating and Number of Parents Selecting Each Additional Facilitator*

| New Facilitator Statement | Rating | *n* |
| --- | --- | --- |
| 1. I had Social Support from family, friends, and/or other parents  of autistic children | 3 | 1 |
| 2. The support service is informed by the views of autistic adults | 3 | 1 |
| 3. The provider taught the adult~~s~~ in my child’s life (e.g. teachers)  helpful strategies | 4 | 1 |
| 4. The support service is/was available online | 3 | 1 |

**Table S10**

*Percentage of Participants Selecting Paired Barrier and Facilitator Statements*

| **Statement #** | **Percent** | | | | | | | | | | | | | | | | | | | | | | | | | | | | | | | | | | | | | |
| --- | --- | --- | --- | --- | --- | --- | --- | --- | --- | --- | --- | --- | --- | --- | --- | --- | --- | --- | --- | --- | --- | --- | --- | --- | --- | --- | --- | --- | --- | --- | --- | --- | --- | --- | --- | --- | --- | --- |
|  | **B1** | **B2** | **B3** | **B4** | **B5** | **B6** | **B7** | **B8** | **B9** | **B**  **10** | **B**  **11** | **B**  **12** | **B**  **13** | **B**  **14** | **B**  **15** | **B**  **16** | **B**  **17** | **B**  **18** | **B**  **19** | **B**  **20** | **B**  **21** | **B**  **22** | **B**  **23** | **B**  **24** | **B**  **25** | **B**  **26** | **B**  **27** | **B**  **28** | **B**  **29** | **B**  **30** | **B**  **31** | **B**  **32** | **B**  **33** | **B**  **34** | **B**  **35** | **B**  **36** | **B**  **37** | **B**  **38** |
| **F1** | 0 | 3 | 3 | 0 | 1 | 3 | 3 | 3 | 2 | 4 | 2 | 2 | 2 | 2 | 3 | 3 | 3 | 4 | 2 | 2 | 3 | 2 | 2 | 1 | 1 | 1 | 3 | 4 | 1 | 3 | 1 | 3 | 1 | 2 | 2 | 1 | 1 | 2 |
| **F2** | 1 | 52 | 55 | 9 | 18 | 52 | 51 | 51 | 34 | 50 | 36 | 18 | 43 | 53 | 38 | 40 | 31 | **68** | 22 | 30 | 43 | 22 | 37 | 22 | 7 | 23 | 58 | **62** | 22 | 43 | 39 | 50 | 32 | 30 | 38 | 39 | 8 | 28 |
| **F3** | 1 | 37 | 35 | 6 | 13 | 34 | 34 | 33 | 22 | 32 | 25 | 10 | 29 | 33 | 19 | 27 | 18 | 44 | 13 | 18 | 27 | 13 | 21 | 12 | 3 | 11 | 36 | 39 | 17 | 26 | 22 | 28 | 20 | 12 | 22 | 25 | 6 | 20 |
| **F4** | 1 | 23 | 24 | 5 | 13 | 20 | 20 | 21 | 12 | 20 | 15 | 8 | 19 | 21 | 15 | 13 | 15 | 29 | 8 | 13 | 15 | 11 | 16 | 11 | 3 | 8 | 20 | 25 | 7 | 19 | 15 | 20 | 13 | 9 | 17 | 14 | 3 | 10 |
| **F5** | 1 | 21 | 22 | 5 | 10 | 19 | 17 | 20 | 10 | 19 | 13 | 8 | 19 | 19 | 12 | 13 | 15 | 28 | 8 | 12 | 15 | 10 | 16 | 10 | 3 | 7 | 21 | 24 | 8 | 17 | 14 | 20 | 12 | 11 | 14 | 12 | 2 | 8 |
| **F6** | 2 | 47 | 47 | 6 | 15 | 42 | 45 | 48 | 25 | 41 | 31 | 13 | 41 | 46 | 27 | 36 | 29 | **62** | 24 | 24 | 36 | 20 | 33 | 17 | 5 | 18 | 53 | 58 | 22 | 37 | 28 | 43 | 27 | 23 | 29 | 27 | 6 | 23 |
| **F7** | 2 | 31 | 32 | 5 | 10 | 29 | 26 | 27 | 15 | 26 | 20 | 10 | 22 | 29 | 18 | 22 | 17 | 42 | 18 | 20 | 27 | 11 | 20 | 15 | 4 | 11 | 31 | 36 | 15 | 24 | 23 | 30 | 20 | 20 | 20 | 19 | 5 | 15 |
| **F8** | 1 | 28 | 28 | 5 | 9 | 26 | 20 | 18 | 11 | 22 | 19 | 6 | 20 | 22 | 13 | 22 | 14 | 35 | 14 | 15 | 22 | 9 | 19 | 10 | 3 | 10 | 27 | 27 | 12 | 22 | 17 | 25 | 17 | 14 | 15 | 16 | 4 | 13 |
| **F9** | 3 | 51 | 50 | 8 | 15 | 47 | 48 | 54 | 27 | 45 | 31 | 15 | 43 | 49 | 29 | 36 | 30 | **67** | 25 | 28 | 40 | 23 | 33 | 21 | 6 | 19 | 56 | **62** | 23 | 41 | 35 | 49 | 29 | 26 | 30 | 30 | 7 | 24 |
| **F**  **10** | 1 | 48 | 46 | 5 | 11 | 41 | 43 | 48 | 25 | 39 | 31 | 15 | 38 | 45 | 27 | 34 | 29 | **61** | 24 | 24 | 37 | 17 | 31 | 17 | 6 | 17 | 52 | 57 | 21 | 35 | 30 | 44 | 27 | 25 | 29 | 28 | 4 | 22 |
| **F**  **11** | 2 | 54 | 53 | 8 | 16 | 48 | 51 | 55 | 31 | 48 | 33 | 17 | 45 | 53 | 30 | 37 | 30 | **69** | 26 | 27 | 41 | 20 | 33 | 20 | 6 | 18 | **60** | **65** | 23 | 45 | 35 | 49 | 30 | 26 | 34 | 35 | 7 | 24 |
| **F**  **12** | 2 | 51 | 52 | 6 | 16 | 47 | 48 | 52 | 29 | 47 | 30 | 17 | 45 | 52 | 30 | 37 | 29 | **68** | 27 | 27 | 38 | 20 | 34 | 21 | 7 | 19 | 59 | **64** | 23 | 44 | 34 | 48 | 30 | 25 | 33 | 31 | 7 | 24 |
| **F**  **13** | 2 | 40 | 44 | 6 | 11 | 41 | 38 | 43 | 28 | 41 | 24 | 15 | 36 | 41 | 29 | 29 | 26 | 55 | 23 | 24 | 33 | 17 | 27 | 17 | 6 | 17 | 50 | 52 | 20 | 37 | 29 | 39 | 23 | 25 | 29 | 26 | 6 | 21 |
| **F**  **14** | 1 | 13 | 16 | 2 | 3 | 13 | 9 | 12 | 6 | 10 | 11 | 4 | 11 | 13 | 8 | 12 | 7 | 21 | 10 | 6 | 14 | 4 | 12 | 7 | 2 | 6 | 15 | 17 | 6 | 12 | 11 | 16 | 11 | 10 | 10 | 10 | 3 | 5 |
| **F**  **15** | 2 | 28 | 31 | 3 | 6 | 24 | 24 | 26 | 13 | 25 | 17 | 10 | 22 | 24 | 15 | 25 | 14 | 40 | 17 | 15 | 24 | 9 | 21 | 14 | 4 | 11 | 33 | 37 | 13 | 24 | 18 | 27 | 18 | 18 | 20 | 19 | 5 | 13 |
| **F**  **16** | 2 | 32 | 31 | 3 | 11 | 28 | 27 | 28 | 15 | 25 | 21 | 10 | 23 | 30 | 17 | 22 | 17 | 42 | 16 | 17 | 24 | 13 | 20 | 17 | 3 | 8 | 36 | 36 | 16 | 27 | 20 | 29 | 20 | 19 | 22 | 19 | 5 | 15 |
| **F**  **17** | 1 | 19 | 16 | 2 | 4 | 17 | 16 | 18 | 10 | 14 | 13 | 6 | 17 | 16 | 12 | 11 | 9 | 26 | 10 | 10 | 17 | 7 | 13 | 8 | 3 | 6 | 20 | 23 | 8 | 15 | 13 | 18 | 10 | 13 | 11 | 10 | 1 | 9 |
| **F**  **18** | 1 | 20 | 21 | 3 | 8 | 22 | 17 | 18 | 12 | 15 | 15 | 6 | 20 | 20 | 12 | 15 | 13 | 26 | 11 | 13 | 17 | 10 | 9 | 9 | 4 | 7 | 20 | 23 | 9 | 16 | 12 | 20 | 13 | 13 | 15 | 13 | 3 | 12 |
| **F**  **19** | 1 | 34 | 33 | 5 | 10 | 28 | 31 | 31 | 16 | 27 | 22 | 8 | 27 | 34 | 17 | 24 | 20 | 45 | 17 | 18 | 26 | 15 | 22 | 14 | 4 | 11 | 36 | 41 | 15 | 22 | 20 | 34 | 21 | 17 | 19 | 18 | 5 | 13 |
| **F**  **20** | 2 | 37 | 34 | 4 | 11 | 33 | 35 | 36 | 17 | 31 | 22 | 10 | 27 | 36 | 20 | 27 | 21 | 48 | 19 | 20 | 31 | 13 | 23 | 17 | 6 | 13 | 40 | 44 | 17 | 28 | 23 | 37 | 24 | 18 | 20 | 21 | 5 | 16 |
| **F**  **21** | 2 | 47 | 46 | 6 | 13 | 41 | 45 | 50 | 24 | 41 | 31 | 12 | 39 | 45 | 27 | 35 | 27 | **61** | 24 | 26 | 38 | 19 | 32 | 18 | 6 | 19 | 52 | 58 | 23 | 34 | 30 | 46 | 28 | 24 | 29 | 28 | 6 | 22 |
| **F**  **22** | 1 | 27 | 27 | 4 | 8 | 25 | 28 | 29 | 17 | 26 | 21 | 11 | 25 | 31 | 17 | 20 | 15 | 36 | 13 | 18 | 24 | 12 | 21 | 11 | 3 | 13 | 31 | 34 | 11 | 22 | 19 | 27 | 16 | 17 | 22 | 21 | 3 | 17 |
| **F**  **23** | 1 | 11 | 13 | 3 | 3 | 13 | 11 | 13 | 7 | 10 | 10 | 6 | 14 | 14 | 8 | 8 | 8 | 18 | 8 | 6 | 13 | 5 | 10 | 8 | 3 | 6 | 15 | 16 | 6 | 9 | 9 | 14 | 8 | 10 | 10 | 9 | 1 | 6 |
| **F**  **24** | 1 | 30 | 27 | 3 | 8 | 29 | 27 | 29 | 15 | 23 | 19 | 9 | 26 | 29 | 19 | 19 | 18 | 39 | 14 | 16 | 24 | 12 | 22 | 15 | 4 | 10 | 34 | 35 | 13 | 24 | 22 | 31 | 20 | 20 | 21 | 21 | 3 | 15 |
| **F**  **25** | 1 | 39 | 38 | 6 | 13 | 36 | 36 | 38 | 22 | 35 | 27 | 13 | 31 | 39 | 24 | 31 | 22 | 52 | 22 | 21 | 31 | 17 | 27 | 15 | 5 | 19 | 42 | 46 | 17 | 31 | 27 | 36 | 25 | 23 | 28 | 27 | 6 | 20 |
| **F**  **26** | 1 | 38 | 37 | 6 | 12 | 34 | 35 | 39 | 22 | 34 | 26 | 10 | 31 | 36 | 22 | 27 | 17 | 48 | 17 | 15 | 25 | 11 | 24 | 13 | 4 | 12 | 44 | 48 | 10 | 28 | 23 | 35 | 20 | 19 | 25 | 26 | 4 | 17 |
| **F**  **27** | 1 | 27 | 27 | 3 | 6 | 24 | 22 | 22 | 15 | 21 | 19 | 7 | 22 | 24 | 13 | 22 | 13 | 37 | 15 | 10 | 19 | 10 | 17 | 11 | 3 | 7 | 29 | 30 | 13 | 22 | 18 | 25 | 18 | 14 | 16 | 15 | 5 | 11 |
| **F**  **28** | 2 | 54 | 52 | 6 | 16 | 48 | 50 | 55 | 30 | 48 | 35 | 17 | 45 | 52 | 31 | 40 | 32 | **69** | 26 | 29 | 43 | 22 | 36 | 21 | 7 | 20 | 59 | **64** | 23 | 42 | 33 | 51 | 31 | 27 | 34 | 34 | 8 | 25 |
| **F**  **29** | 1 | 22 | 22 | 4 | 7 | 19 | 15 | 15 | 12 | 16 | 11 | 7 | 17 | 18 | 10 | 15 | 8 | 27 | 13 | 13 | 18 | 7 | 14 | 8 | 3 | 8 | 21 | 24 | 9 | 16 | 12 | 19 | 13 | 12 | 12 | 13 | 3 | 9 |
| **F**  **30** | 1 | 20 | 20 | 2 | 5 | 16 | 10 | 13 | 8 | 14 | 11 | 5 | 15 | 14 | 10 | 13 | 9 | 24 | 10 | 10 | 15 | 6 | 13 | 9 | 3 | 6 | 19 | 20 | 8 | 15 | 10 | 17 | 11 | 10 | 10 | 8 | 3 | 8 |
| **F**  **31** | 1 | 22 | 21 | 4 | 6 | 20 | 13 | 13 | 11 | 15 | 12 | 6 | 13 | 16 | 10 | 15 | 8 | 29 | 10 | 10 | 17 | 6 | 13 | 10 | 3 | 5 | 22 | 22 | 11 | 16 | 13 | 20 | 13 | 11 | 9 | 13 | 4 | 10 |
| **F**  **32** | 2 | 35 | 34 | 3 | 9 | 34 | 33 | 36 | 15 | 29 | 22 | 11 | 27 | 34 | 19 | 26 | 19 | 49 | 17 | 18 | 31 | 13 | 23 | 17 | 7 | 14 | 38 | 45 | 15 | 24 | 23 | 36 | 21 | 20 | 21 | 22 | 5 | 16 |
| **F**  **33** | 1 | 39 | 41 | 4 | 12 | 39 | 36 | 41 | 21 | 34 | 28 | 10 | 33 | 41 | 22 | 31 | 24 | 55 | 21 | 20 | 31 | 16 | 27 | 17 | 6 | 13 | 45 | 50 | 20 | 31 | 25 | 38 | 25 | 21 | 28 | 25 | 7 | 20 |
| **F**  **34** | 2 | 30 | 31 | 4 | 11 | 28 | 27 | 31 | 14 | 24 | 20 | 9 | 24 | 29 | 13 | 20 | 16 | 43 | 15 | 15 | 23 | 11 | 18 | 14 | 4 | 8 | 36 | 39 | 13 | 23 | 19 | 30 | 19 | 18 | 18 | 18 | 4 | 13 |
| **F**  **35** | 2 | 39 | 36 | 6 | 12 | 34 | 32 | 35 | 17 | 30 | 24 | 12 | 29 | 36 | 19 | 27 | 22 | 50 | 20 | 20 | 31 | 14 | 25 | 16 | 5 | 14 | 39 | 45 | 16 | 27 | 24 | 36 | 23 | 18 | 23 | 23 | 6 | 19 |
| **F**  **36** | 1 | 27 | 24 | 2 | 6 | 24 | 22 | 22 | 13 | 20 | 15 | 8 | 19 | 22 | 13 | 20 | 14 | 34 | 14 | 11 | 21 | 10 | 14 | 10 | 3 | 8 | 27 | 29 | 12 | 17 | 16 | 25 | 16 | 14 | 13 | 14 | 4 | 13 |
| **F**  **37** | 2 | 34 | 33 | 6 | 13 | 34 | 30 | 32 | 20 | 31 | 22 | 13 | 30 | 31 | 22 | 22 | 21 | 43 | 20 | 22 | 31 | 15 | 25 | 17 | 3 | 12 | 35 | 39 | 20 | 29 | 25 | 37 | 25 | 22 | 22 | 22 | 5 | 15 |
| **F**  **38** | 1 | 42 | 41 | 5 | 14 | 36 | 36 | 40 | 24 | 35 | 27 | 13 | 34 | 41 | 28 | 31 | 24 | 52 | 19 | 20 | 31 | 15 | 27 | 15 | 4 | 17 | 44 | 48 | 18 | 32 | 24 | 36 | 25 | 20 | 29 | 27 | 6 | 18 |

*Note.* **Bold values** indicate that 60% or more of participants selected both statements
